# Recent Achievements in Dyes Removal Focused on Advanced Oxidation Processes Integrated with Biological Methods

**DOI:** 10.3390/molecules26040870

**Published:** 2021-02-06

**Authors:** Stanisław Ledakowicz, Katarzyna Paździor

**Affiliations:** Department of Bioprocess Engineering, Faculty of Process and Environmental Engineering, Lodz University of Technology, Wólczańska 213, 90-924 Łódź, Poland

**Keywords:** dyes and pigments, textile wastewater, decolorization, AOPs, biological processes

## Abstract

In the last 3 years alone, over 10,000 publications have appeared on the topic of dye removal, including over 300 reviews. Thus, the topic is very relevant, although there are few articles on the practical applications on an industrial scale of the results obtained in research laboratories. Therefore, in this review, we focus on advanced oxidation methods integrated with biological methods, widely recognized as highly efficient treatments for recalcitrant wastewater, that have the best chance of industrial application. It is extremely important to know all the phenomena and mechanisms that occur during the process of removing dyestuffs and the products of their degradation from wastewater to prevent their penetration into drinking water sources. Therefore, particular attention is paid to understanding the mechanisms of both chemical and biological degradation of dyes, and the kinetics of these processes, which are important from a design point of view, as well as the performance and implementation of these operations on a larger scale.

## 1. Introduction

Dyes and pigments are colorants that give a color to a material, making it more attractive. The major difference between dyes and pigments is the particle diameter—dyes are much smaller than pigments—and solubility in liquids—dyes are soluble and penetrate into the textile or a material, while pigments are insoluble and are suspended in liquid, forming a thin film and painting the surface of the material. Dyeing is believed to have originated in Neolithic times, some 4000 to 9000 years ago. The coloring materials used were obtained from natural sources such as plants, insects, and shells. Plant-based dyes such as woad, indigo, saffron, madder, alkanna, henna, brazilwood, red sandalwood, safflower, and logwood were used for dyeing. Though natural dyes are ecofriendly, protective to skin, and aesthetically pleasing, they have very poor bonding with textile fiber materials, which necessitates mordanting with metallic mordants (substances capable of combining with a dye and the material, increasing the binding of the dye, e.g., chrome alum); however, most of these are not ecofriendly. After the synthesis of mauveine by H. Perkin in 1856 and the subsequent commercialization of synthetic dyes, they replaced natural dyes [1].

Nowadays, there are about 8000 different synthetic dyes, listed in the Color Index (C.I.), under 40,000 trade names [2]. Factors considered in dye selection include fastness to light, reaction to washing and rubbing, and the cost of the dyeing process. The global dye and pigment market was valued at USD 33.2 billion in 2019 [2] and is expected to reach USD 49.1 billion by 2027. Around 70 million tons of synthetic dyes are produced annually for the textile industry worldwide, of which nearly 10% of the dyestuff is discharged to the environment as process wastewater [3], because even up to 50% of the dyes used are not fixed to the textile fibers, but persist as pollutants in the liquid phase [4]. It is worth mentioning that dyes are stable and difficult to degrade due to their complex aromatic structure and synthetic origin. Moreover, the textile industry’s effluents contain detergents, surfactants, dispersants, levelling agents, toxic organics (phenols), chlorinated compounds (AOX), sulphide, and formaldehyde, which may be added to improve dye adsorption onto the fibers, as well as inhibitory compounds, grease and oil, and many other compounds depending on the particular textile process such as scouring, desizing, mercerizing, bleaching, dyeing, printing, and finishing.

The complex textile effluent is a cause of a significant amount of environmental degradation and human illnesses. Many synthetic dyes and their metabolic intermediate products are found to be toxic, mutagenic, and carcinogenic. The major toxic effects of azo dyes are caused by aromatic amines generated after their biodegradation [5]. About 40% of globally used colorants contain organically bound chlorine, a known carcinogen [6]. All the organic materials present in the wastewater from the textile industry are of great concern in water treatment. Discharge of the colored effluent into streams and rivers results in the depletion of dissolved oxygen, causing anoxic conditions that are lethal to aquatic organisms.

The textile industry is one of the most water consuming industries, and requires a high demand of water for its various sectors, needing between 80 and 400 L to produce 1 kg of textiles. The average ratio of water to textile production is 200 tons to 1 ton in most fabric manufacturing facilities [7]. The daily water consumption of an average sized textile mill with a production of about 8000 kg of fabric per day is about 1.6 million liters, 16% of which is consumed in dyeing and 8% in printing. The overall water consumption of yarn dyeing is about 60 L per kg of yarn. The dyeing section contributes to 15–20% of the total wastewater flow [6]. The most notable environmental impact is wastewater discharge (115–175 kg O_2_ of Chemical Oxygen Demand (COD) per ton of finished product, a wide range of organic chemicals, color, salinity, and low biodegradability [8]).

Various physical, chemical, and biological methods such as adsorption, photolysis, chemical precipitation, chemical oxidation and reduction, electrochemical precipitation, and membrane processes as well as their combinations have been employed for the removal of dyes from highly polluted wastewater. However, many of these methods have considerable drawbacks, including high cost, or harsh reacting conditions or, as with adsorption techniques, are nothing more than the transfer of pollutants from the liquid phase to the solid. In contrast to these techniques, biological methods of treating textile wastewater are much cheaper, efficient, and environmentally friendly. Specifically, two biodegradation methods, microbial and enzymatic systems, have been shown to be useful approaches in industrial textile effluent treatment [9,10]. However, the conventional biological processes do not always provide satisfactory results, especially for industrial textile wastewater, since many of the organic substances emitted from dyehouses are toxic or resistant to biological treatment. Therefore, the only feasible option for such biologically persistent wastewater is the use of a combination of biodegradation and advanced oxidation processes (AOPs), widely recognized as highly efficient treatments for recalcitrant wastewater.

Recently, Miklos et al. [11] provided a critical review of different established and emerging AOPs. To facilitate a comparison of energy efficiency, the authors critically compared the AOPs and based on electrical energy per order (EEO) values classified the processes in the following descending order: O_3_, O_3_/H_2_O_2_, O_3_/UV, UV/H_2_O_2_, UV/persulfate, UV/Cl_2_, electron beam, representing median EEO values lower than 1 kWh m^−3^/order, and photo-Fenton, plasma, and electrolytic AOPs of significantly higher EEO values. A similar ranking system to numerically score the performance of various AOPs (e.g., ozonation, UV irradiation, photocatalysis, Fenton reaction) in several categories of parameters under engineering, environmental, and socioeconomic components was published by Fast et al. [12]. From this preliminary assessment, it was noted that H_2_O_2_/O_3_ (perozonation or peroxone) presented the highest average ranking, with other processes showing comparable performance, while TiO_2_ photocatalysis received the lowest ranking. Additionally, taking into account the fact that light penetration in dye-colored solutions is low, we decided not to include photocatalytic processes in this review, due to their low chance of implementation in industrial practice.

In order to avoid unnecessary expenditure of chemicals and energy, thereby lowering the operating cost, the application of biological oxidation either as a pre-treatment or as a post-treatment was proposed [13]. The chemical pre-treatment acts as partial oxidation of the biologically persistent part to produce biodegradable reaction intermediates, while in the opposite direction, the highly biodegradable part of the wastewater is first eliminated in a biological way and then the recalcitrant contaminants are degraded in AOPs post-treatment.

Taking these considerations into account, we decided to limit this review to integrated AOPs processes with biological methods that have the best chance of practical implementation of the treatment of colored wastewater of the textile industry. Although we published a review of the existing and emerging technologies in the combination of AOPs and biological processes 3 years ago, this time we decided to focus on novelties of the past 3 years, but not only on industrial textile wastewater, which this review was about [14]. Moreover, particular attention was paid to understanding the mechanisms of both chemical and biological degradation of dyes, the kinetics of these processes, as well as the performance and the implementation of these operations on a larger scale, because it is extremely important to know all the phenomena and mechanisms that occur during the process of removing dyestuffs and the products of their degradation from wastewater to prevent their penetration into drinking water sources. Moreover, taking into account that this review covers two fields of biology and chemistry, we decided to introduce the readers to the fundamentals of both chemical and biological processes applied to textile wastewater treatment, and provide the most important achievements in these areas. In addition, from the point of view of designing reactors for the treatment of colored textile wastewater, it is important to know the kinetics of the process, and therefore we paid attention to this kinetic aspect of both chemical and biological dye degradation processes.

This is important especially due to the fact that methods and technologies for wastewater treatment and water recovery are constantly being modified to improve their effectiveness.

## 2. Classifications of Dyestuffs and Characteristics of Textile Wastewater

It is estimated that over 100,000 commercially available dyes exist [15]. They are classified according to their application and chemical structure [16]. The dye classification groups based on chemical structure are: azo, anthraquinone, indigo, phthalocyanine, sulfur, nitro, and nitroso, etc., and according to application, the groups are: reactive, dispersed, acid, basic, direct, and vat [3,17]. They contain a group of atoms responsible for the dye color, called chromophores [18]. Additionally, they may have also auxochromes—an electron withdrawing or donating substituents that cause or intensify the color of the chromophores [18]. The most common chromophores are azo, carbonyl, methane, nitro, and quinoid groups [16], while among the auxochromes, the most important are amine, carboxyl, sulfonate, and hydroxyl groups [18]. The azo dyes are the most used textile dyes, as azo chromophores give the possibility to obtain the widest range of colors [19]. The dye’s degradation is dependent on its structure and properties. Moreover, binding the dye to the fabric requires the use of many auxiliaries that are also connected to the type of dye [7].

In conventional dyeing by means of reactive dyes, the fixation rate is often less than 80%, resulting in the need to remove that 20% (which is not fixed) from the fabric. Dye fixation is usually carried out in the presence of an alkali. Due to the addition of an alkali, the dye migrates from outside of the fiber to the inside of the fiber and forms a covalent bond with the fiber. Therefore, textile wastewater has a high pH value, high concentration of suspended solids, chlorides, nitrates, and metals (sodium, lead, copper, chromium, iron), and high Biochemical Oxygen Demand (BOD) and COD value. The discharge of a huge amount of colored wastewater containing 100–150g L^−1^ of NaCl or Na_2_SO_4_ results in serious water pollution and land salinization [20].

**Figure 1:**
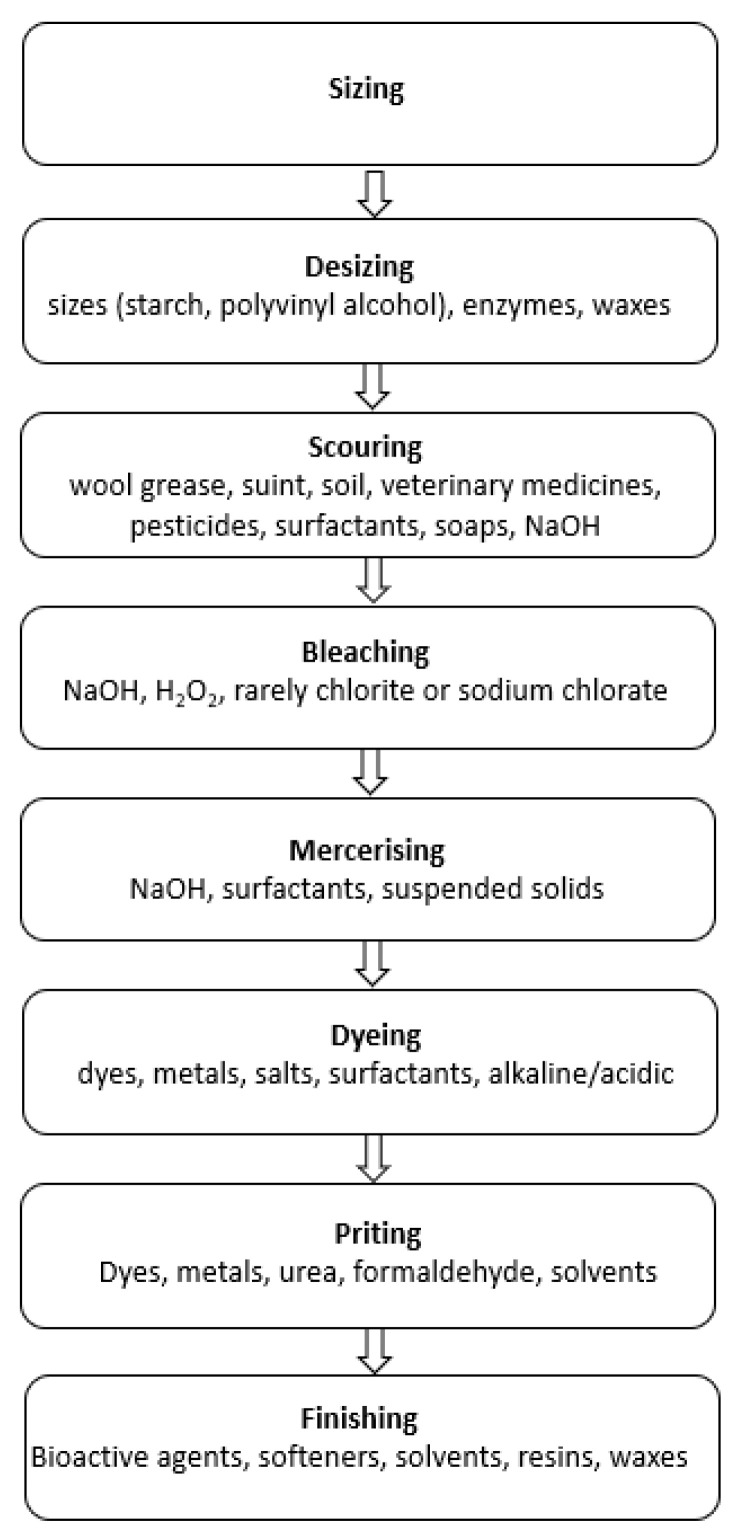
The most common pollutants generated in wet processes [14,21,26].

Wet processes—finishing processes such as washing, bleaching, mercerizing, dyeing, functional finishing and coating—are mostly responsible for the water demand, as well as the wastewater production in this industry branch [21,22]. As can be seen in Figure 1, the textile effluents are loaded not only by dyes but also by the significant amounts of other chemicals. Apparently, dyeing machines are the main source of the textile wastewater containing dyes. Most often, they work in batch mode, generating several effluents differing significantly—Table 1 shows fluctuations in the selected parameter values for reactive dyeing of cotton. Some of them may be characterized as low-loaded by contaminants (e.g., from rinsing). Among the more polluted streams (effluents after washing, acidification and dyeing), the most problematic is the dyeing one, as it is not only heavily loaded by organic chemicals (e.g., dyes), but also by electrolytes (Table 1). As a result, the values of the BOD_5_/COD ratio for this stream are very low (Table 1)—in the range of non-biodegradable wastewater [23]. The wastewater biodegradability may be estimated on the basis of: BOD_5_/COD ratio, average oxidation state (AOS), bacterial growth rate, oxygen uptake rate tests or toxicity (most often as the EC50 value towards the bacteria *Vibrio fisheri*) [24]. The simplest method is the calculation of the BOD_5_/COD ratio. It is assumed that wastewater with BOD_5_/COD below 0.2 is nonbiodegradable. BOD_5_/COD between 0.2 and 0.6 means that the solution is biodegradable with selected microorganisms, while BOD_5_/COD above 0.6 characterizes biodegradable wastewater [25].

The literature characteristics of textile wastewater are usually averaged for the general effluent of the textile plant. Yaseen and Scholz [26] presented a critical review of the currently available literature regarding typical and real characteristics of the textile effluents; however, the authors concentrated on the scattered information relating to simulated textile wastewater. By the way, it should be noted that in the literature on dye removal, many experimental studies were carried out, if not in single dye or model dye mixtures, then on simulated textile wastewater, which, as it was shown in [28] by Bilińska et al., significantly differs from the treatment of real industrial wastewater. The typical values of parameters such as specific conductivity, pH, COD, BOD, TC, TOC, total phosphorus, total nitrogen and chloride content characterized real industrial textile wastewater from several technological operations in a dyehouse were determined, and based on the conducted survey, the biodegradability of the tested baths was determined according to BOD_5_/COD, N/P, BOD_5_/N/P, and toxicity towards activated sludge microorganisms [27]. On this basis, a conclusion was drawn that there is the need to separate dye effluent streams according to their biodegradability. This is in accordance to recommendations of European Commission Integrated Pollution Prevention and Control (IPPC) Reference Document on Best Available Techniques for the Textiles Industry July 2003 [29] and the revised BREF document (December 2019) [30]—decentralized treatment on site of selected, segregated single wastewater streams. Generally, textile wastewater can be divided into low-loaded, easily biodegradable streams and high-loaded streams (COD above 5000 mgO_2_ L^−1^), which are usually highly saline and less biodegradable [29].

The BOD_5_/COD ratio for dyes varies in a very wide range—between 0 and 0.8 [31,32]. A lot of dyes have BOD_5_ equal to 0 (for example, Reactive YellowKD-3G, Reactive Red 24, Cationic BlueX-GRL, [32]). On the one hand, this may be connected to the fact that they are synthetic, xenobiotic substances, but on the other, some of the dyes are just toxic to microorganisms or other trophic levels of aquatic life [33,34,35]. In many cases, a dye’s toxicity is connected to the heavy metals content in molecules. Additionally, absorption of the light by dye molecules causes problems for photosynthetic aquatic plants and algae [36]. Moreover, some of them are mutagenic or cytotoxic [37,38].

One of the major contaminants in wastewater treatment can be identified as the metals and their complexes which are either dissolved or present in other forms in real water effluents. Cadmium, lead, zinc, and chromium, etc., are the most toxic metals found in wastewater during manufacturing processes in textile industries [39].

Depending on the dye structures and presence of functional groups, dyes differ in the level of toxicity. Some azo dyes can be mutagenic without being cleaved into aromatic amines. A drastic example includes dyes containing benzidine in their composition (currently prohibited for use in the EU), which induced various human and animal tumors. Another azo dye component, p-phenylenediamine, is an allergen. Many azo dyes and their reductively cleaved products as well chemically related aromatic amines are reported to affect human health causing allergies [40].

Generally, not only do textile dyes cause many threats for the environment, but they also occur together with their by-products and other pollutants in industrial effluents. As a result, textile dye treatment must be flexible and take into account the interactions between different components (e.g., chlorides and oxidants).

## 3. Processes Used in the Textile Dyes Removal

All methods used in dye degradation may be divided into chemical, physico-chemical, and biological [41]. Table 2 shows current trends in the investigations on most of the processes implemented to dye degradation based on the literature within the last two years. It must be stressed that the economically and environmentally viable treatment systems consist of combined processes.

As seen from Table 2, there are numerous existing tried and tested methods to accomplish dye removal. Only in 2019, Deng et al. [156] reviewed 42 papers on textile wastewater treatment technologies organized by physico-chemical, biological, and combined processes. There is continuous progress in the number of articles on this subject. However, Katherasan et al. [157] stressed that it is difficult to decide on a single technique that resolves the prevailing dye effluent issue, due to lack of information on the efficiency of dye removal methods; moreover, most of them have a common disadvantage, which is the generation of secondary pollution to the environment. These authors suggested the usage of a combined adsorbent, as it is envisioned that this technique has better efficiency and is able to remove dyes at a faster rate. However, they did not answer what to do with the used adsorbents. As was mentioned in the Introduction, we decided to focus on the integrated AOPs with biological methods that have the best chance of industrial implementation.

As was mentioned in the Introduction, Fast et al. [12] ranked various AOPs (e.g., ozonation, UV irradiation, photocatalysis, Fenton reaction, and integrated processes) applied in the removal of emerging contaminants. TiO_2_ photocatalysis received the lowest ranking, which was directly related to the extremely high electrical costs (USD 2285.02 m^−3^), that are significantly higher than other processes (e.g., O_3_—USD 0.32 m^−3^, Fenton—USD 3.77 m^−3^). However, these rankings are not an absolute indication of advantage, because some other parameters must be considered as more influential. Economic and social parameters caused the most significant variation in scores due to electricity costs.

## 4. Mechanism and Kinetics of AOPs

Advanced oxidation processes (AOPs) are those groups of technologies that lead to the generation of the hydroxyl radical (**·**OH) or others such as the sulfate radical SO_4_^−^. The hydroxyl radical is the second recognized oxidant with the highest oxidative power after the fluorine and its redox potential E_0_ = 2.8 V is higher than other oxidizing agents, such as sulphate anion radicals SO_4_^−^, ozone O_3_, hydrogen peroxide H_2_O_2_, and others. Glaze et al. [158] were the first to generate hydroxyl radicals (**·**OH) in sufficient quantities to affect water purification and defined the term “Advanced Oxidation Processes”. Since the 1990s, the development of AOPs has grown and they include many methods to produce hydroxyl radicals and other reactive oxidant species, including superoxide anion radicals O_2_^•−^ and singlet oxygen O_2_(^1^Δ_g_). Hydroxyl radicals are produced with the help of one or more primary oxidants (e.g., ozone, hydrogen peroxide, oxygen) and/or energy sources (e.g., ultraviolet light) and with catalysts (e.g., titanium dioxide).

According to Harvey and Rutledge [159], chemistry in AOPs could be essentially divided into three stages:

1. Formation of **·**OH;
2. Initial attacks on target molecules by **·**OH and their breakdown into fragments;
3. Subsequent attacks by **·**OH until ultimate mineralization.

The mechanism of **·**OH generation (Stage 1) highly depends on the sort of AOP technique that is used.

### 4.1. Ozone-Based AOPs

In the case of ozonation, the mechanism of **·**OH generation is based on O_3_ decomposition in a chain of reactions, initiated by HO^−^ ions according to the model proposed by Staehelin et al. [160] or by HO_2_^−^ ions according to the model proposed by Tomiyasu et al. [161].

The reaction sequence (initiation, propagation, and termination) can be summarized according to Beltran [162] as follows:

O_3_ + HO^−^ → HO_2_^−^ + O_2_ *(reaction between O_3_ and a hydroxyl ion)*

O_3_ + HO_2_^−^ → HO_2_**^·^** + O_3_^−**·**^ *(a second O_3_ reacts with the HO_2_^−^ to produce the ozonide radical)*

HO_2_**·⇄** O_2_^•−^ + H^+^ (*formation of superoxide ion* O_2_^•−^*, from which the transfer of electron to* O_3_ *causes the formation of* O_3_^−**·**^ *and subsequently* **·**OH)

O_3_^−**·**^ + H^+^ → HO_3_**^·^** *(this radical gives **·**OH upon protonation)*

HO_3_ → **·**OH + O_2_

Thus, ozone decomposition may be significantly promoted in a basic solution (7 < pH < 10), whereas it may be less effective in an acidic solution (pH < 3). In high pH, ozone is able to form the more powerful nonselective oxidants of hydroxyl radicals (E_0_ = 2.8 V) compared to its own redox potential (E_0_ = 2.08 V), which is still high enough to break down the complex aromatic rings of dyestuffs, resulting in the decolorization.

O_3_ + 2H^+^ + 2e → O_2_ + H_2_O

In an acidic environment, ozone can react with reactive dyes more effectively than with other types, such as sulfur, acid, or disperse dyes; however, the direct oxidation is relatively slow compared to hydroxyl radical oxidation.

The initial attacks of **·**OH radical on the target molecule (Stage 2) is such a fast reaction that the pulse radiolysis technique should be employed, as was demonstrated by Perkowski et al. [163] in the decomposition of anthraquinone dye Acid Blue 62. The rate constant of the reaction of this dyestuff with **·**OH reaches 1.0 × 10^10^ M^−1^s^−1^. The product of the reaction of the radical **·**OH and dyestuff participates in consecutive reactions, causing its decomposition with the cleavage of the aromatic ring. Generally, **·**OH radicals behave like a highly reactive electrophile and two types of initial attack are supposed to be addition and hydrogen abstraction. However, in water, there are several compounds that are capable of the initiation and promotion but also inhibition of the radical chain reaction process. The initiators (OH^−^, H_2_O_2_, formate, humic substances) can induce the formation of superoxide ion O_2_^•−^ from an ozone molecule. The inhibitors (CH_3_COO^−^, HCO_3_^−^/CO_3_^2−^, humic substances) are compounds capable of consuming hydroxyl radicals. The reaction of hydroxyl radicals is not selective. They react rapidly with the primary radical traps, carbonates, bi-carbonates, and tert-butanol. The rate constants are only slightly lower than those of **·**OH with organic compounds (for HCO_3_^−^ kinetic constant is 1.5 × 10^7^ M^−1^s^−1^). This is the reason why the presence of radical scavengers in water can cause the total inhibition of the free radical chain reaction.

Currently, there is no consensus on the detailed mechanisms in Stage 3; however, very few researchers proposed the mechanism of dye decomposition until complete mineralization to CO_2_ and H_2_O. Perkowski et al. [164] proposed a kinetic model of the decolorization process of an aqueous solution of the anthraquinone dye Acid Blue 62, adapting the mechanism of decolorization by irradiation proposed by Hashimoto et al. [165] with some changes to a general description of the kinetics of a radical **·**OH reaction generated in AOPs such as ozonation or H_2_O_2_/UV. Using the Bodenstein quasi-steady state approximation, the rate equation was derived in the form of the dependence of the discoloration degree on the concentration of the dye and oxidizing agent, either O_3_ or H_2_O_2_. The kinetic parameters of the decolorization rate equation were identified. The experimental data were compared with theoretical calculations and a relatively good agreement of the comparison was obtained.

Such a rigorous approach to modelling the kinetics of the decolorization process is rarely found in the literature. The decolorization rate mostly follows pseudo-first order kinetics with respect to dye concentration, e.g., Yang and Yuan [166] and Rekhate and Shrivastava [167], or very rarely an irreversible second order reaction e.g., Zhang et al. [168], who applied Danckwerts surface renewal theory to specify the gas–liquid mass transfer of gaseous O_3_ and to determine the second order reaction rate constant. In order to avoid O_3_ mass transfer limitation, Bilińska et al. [169] investigated the kinetics of Reactive Black 5 (RB5) ozonation in a liquid–liquid system (gaseous O_3_ was previously absorbed in water) under acidic solution (pH 1.88–6.1). The proposed kinetic model, considering ozone self-decomposition, consisted of a set of three differential equations (for O_3_, dyestuff, and colorless by-product) which was integrated numerically. A solution of a non-linear inverse problem allowed for the identification of the kinetic constants based on experimental data. The values of the rate constants for the reaction RB5 and O_3_ were calculated directly from experimental data, while for the by-products reaction with O_3_, the values were calculated from the model optimization with MATLAB computing script. It was concluded that the by-product appeared when RB5 began to decompose and it was subsequently oxidized to another by-product; however, both RB5 and the by-product did not decompose entirely.

The decolorization of the dyes undergoes a fast reaction, since the chromophore groups with conjugated double bonds, which are responsible for color, can be easily broken down by ozone either directly or indirectly, forming smaller, usually colorless molecules. Therefore, the decolorization extent is mostly near to 100%, but the nature of the chromophore group has a great impact on the efficiency of decolorization [170]. However, the by-products formed due to dye oxidation are very stable, because they still contain aromatic rings and therefore the COD removal efficiency gradually decreased with an increase in the initial dye concentration.

Castro et al. [171] showed that ozone oxidation was very efficient in the degradation of Reactive Orange 16 (RO16) azo dye. Short ozonation times (5 min) were sufficient to achieve high color removal (>97 %) for dye solutions containing up to 100 mg RO16/L. Despite the high color removal capability, the process was not as effective for Total Organic Carbon (TOC) removal (max. TOC removal was 48 %), leading to incomplete mineralization. Eleven intermediate organic compounds resulting from ozone treatment of RO16 solution were identified by LC/MS analyses at different contact times. The identification of ozonation by-products at different contact times enabled a better understanding of the reaction mechanism and the scheme of possible reaction mechanism was postulated. In the first step of the reaction, ozone reacts through an electrophilic reaction, attacking the azo (−N=N−) group, and a fast discoloration was observed. The cleavage of C-N and C-S bonds and further oxidation led to the production of benzene-diol, benzene-tetrol, benzoquinone, hydroxynaphthalene, nitrosonaphtalene, and other derivatives. They observed also that short ozonation time (2 min) led to toxicity decrease, while longer ozonation times caused the increase in the toxicity towards *Vibrio fisheri*. It is in agreement with the results obtained by Dias et al. [35].

As shown, ozonation alone is not an efficient method for treatment of the textile effluent due to low mineralization rate of the refractory by-products as evidenced by the low degree of TOC reduction. To improve ozonation efficiency, it is necessary to find a catalyst with a high degree of activity.

The catalytic ozonation process is an efficient, easy-to-operate, and cost-effective AOP. The most comprehensive review on catalytic ozonation processes and methods aimed at ozonation enhancement was presented by Kasprzyk-Horden et al. [172] and Nawrocki [173], who pointed out a number of controversies in the research on catalytic ozonation and identified some typical experimental errors that are partly responsible for such a situation. In both homogeneous and heterogeneous modes of catalytic ozonation, a variety of solids including metals (Fe(II), Ni(II), Zn(II), Mn(II) and Cr(III)) and metal oxides (Al_2_O_3_, TiO_2_, Fe_2_O_3_, MnO_2_) have been evaluated as catalysts to improve the efficiency of ozonation. Other metals (Cu., Pt, Pb, Pd, Ag, Co, Ru, Ir, Rh, Re) have also been examined as a catalyst [174]. The direct activity of the metal cation on the ozone molecule was reported as a driving force of chain reactions leading to ozone self-decomposition. Two routes of catalytic activity have been proposed for metals on the support. One is the adsorption of the organic molecule on the catalyst active center and then its oxidation by ozone or **·**OH, while the second is ozone decomposition by electron transfer through a reduced or oxidized metal site on the surface of supporting material. The main factors that affect the efficiency, ozone decomposition, and radical formation rate in catalytic ozonation are the type and surface characteristics of catalysts. Asgari et al. [174] synthesized and characterized a new catalyst, a carbon-doped magnesium oxide (C-MgO) doped on an eggshell membrane powder. They demonstrated that using this new catalyst, the degradation and mineralization efficiency of the real textile wastewater in the ozonation process effectively increased—during short reaction times (10 min), 93.7% removal of dye and 78% of TOC was achieved. As shown by Khamparia and Jaspala [175] in their critical review, a large number of studies can be found in the literature, but most of them are limited only to laboratory experiments on synthetic wastewater. So, the high degree of industrial textile wastewater mineralization like Asgari et al. [174] has rarely been achieved.

Nakhate et al. [176] used copper-doped zinc oxide (Cu-doped ZnO) as a catalyst to facilitate degradation of real textile wastewater by ozonation. At the pilot scale installation, they removed 89% of COD within 30 min of reaction. Reduction of COD by catalytic ozonation increased performance six-fold compared with single ozonation. They concluded that the catalytic ozonation resulted in savings in energy consumption during the process, being three times more energy-efficient than single ozonation. Therefore, wastewater treatment uses an approach called catalytic ozonation, which is a promising wastewater treatment solution that is more efficient and effective compared to treatment technologies that have been widely installed in the textile industry.

The reaction of ozone with H_2_O_2_ (peroxone or perozonation) gives rise to **·**OH radicals, so is one of the AOPs that allows one to produce highly reactive **·**OH radicals for pollutant abatement at relatively low cost when compared to other AOPs. Two mols **·**OH are formed per one mol of H_2_O_2_ and two mols of ozone according to overall reaction:

H_2_O_2_ + 2 O_3_ → 2 **·**OH + 3 O_2_

This has been the commonly accepted reaction equation, but recently some modifications were suggested, when it became apparent that the **·**OH yield is only one-half of that given by the stoichiometry in reaction [177]. The new mechanism of the peroxone process proposed the formation of an ozone adduct to hydrogen peroxide anion HO_2_^−^, HO_5_^−^ that decomposes into HO_2_^•^ + O_3_^•−^ and 2O_2_ + OH^−^, the latter accounting for the low **·**OH efficiency. The individual success of H_2_O_2_ and O_3_ may be limited, but the efficiency can be significantly increased if these compounds are merged into one process. This combination of two oxidants can be advantageous in dye removal, such as during the degradation of the compounds that do not absorb UV well. Furthermore, H_2_O_2_/O_3_ has an advantage over photo catalytic processes because of the lack of related equipment and maintenance, which can reduce energy requirements. However, it can be difficult to maintain the proper operating conditions, including chemical dosages and pH level. The actual required ozone dosage is larger than that estimated from stoichiometry. An excess of H_2_O_2_ can cause the quenching of hydroxyl radicals. Residual H_2_O_2_ can also disrupt the proper functioning and reaction of hydroxyl radicals. There are some examples in the literature of applying the peroxone process for decolorization of textile wastewater, e.g., the colour removal efficiency using H_2_O_2_ alone was found to be 23% for a 100 ppm dye solution. Ozone, when combined with H_2_O_2_, improved the efficacy of colour removal from 72 to 90% for a 100 ppm dye solution [178]. The degradation of crystal violet (CV) was investigated by different oxidation processes [79]. The results show that all processes were capable of decolorization. Among these processes, peroxone, with efficiency of about >90%, was more effective than the others. However, the CV degradation products by the peroxone process were more toxic towards *Escherichia coli* than the parent compound.

### 4.2. Fenton Reaction

In 1894, H.J.H. Fenton [179] performed a reaction with iron ions and oxidizing agents. He observed a higher oxidative capacity of the mixture in comparison to its components. Even though the Fenton reaction was initially formulated for Fe(II) and H_2_O_2_, many redox-active metals such as Cu, Mn, and Ni also display Fenton-like reactions.

According to Wang [180], the mechanism of the Fenton process can be represented as follows:

Fe^2+^ + H_2_O_2_ → Fe^3+^ + **·**OH + OH^−^ (*fast generation of **·**OH radicals* k = 40–80 M^−1^s^−1^)

Fe^3+^ + H_2_O_2_ → Fe^2+^ + HO_2_^•^ + H^+^ *(slow* Fe^3+^ *to* Fe^2+^ *reduction kinetics* k = 0.001–0.01 M^−1^s^−1^)

RH + **·**OH → R^•^ + H_2_O (*RH is organic pollutant decomposed by H-abstraction* k = 10^9^ M^−1^s^−1^)

Low pH conditions are required for the Fenton reaction—the optimal pH levels are usually between 2 and 4. If the pH is too low, the scavenging of hydroxyl radicals can increase, but if the pH is too high, the oxidation potential and degradation rates will decrease. Fenton’s reagent was employed by Ledakowicz et al. [181] in the decolorization of aqueous solutions of one of three dyestuffs (Acid Red 27, Reactive Blue 81, Acid Blue 62). The decolorization with Fenton’s reagent was found to be simple and fast. In order to determine the reaction kinetics of the decolorization, the stopped-flow technique under pseudo-first order conditions was used. Experiments were carried out at pH 2, with an excess of ferrous salts (FeCl_2_∙4H_2_O or FeSO_4_∙7H_2_O). The rate constants of the decolorization determined were in the same order of magnitude: 90 to 100 M^−1^s^−1^ for FeSO_4_∙7H_2_O/H_2_O_2_, and 40 to 50 M^−1^s^−1^ for FeCl_2_∙4H_2_O/H_2_O_2_ systems. The difference between the rate constants for both ferrous salts indicates that the Fenton’s reaction may proceed via different mechanisms. In the case of FeCl_2_∙4H_2_O/H_2_O_2_, it is likely that Cl_2_^−^ anion-radicals (E_0_ = 2.1 V) are involved due to the reactions:

**·**OH + Cl^−^ → CIOH^−^

CIOH^−^ + H^+^ → H_2_O+Cl**^∙^**

Cl + Cl^−^ → Cl_2_^−^

Recently, the stopped-flow technique was used to study the decolorization kinetics of methylene blue and rhodamine B with Fenton reagent [182]. The combined pseudo first order model fits the decrease in both methylene blue and rhodamine B with very high regression coefficients. The combined pseudo first order model was proposed based on the two-stage mechanism that two parallel reactions with Fe^2+^ and Fe^3+^ are responsible for dye degradation, whereas the pseudo-first order and pseudo-second order models exhibited poor correlation.

Regarding the thermodynamics of decolorization with Fenton’s reagent, most publications [183,184,185] postulate that this process is spontaneous (negative Gibbs free enthalpy values) and endothermic (positive enthalpy values). However, one publication [186] in the case of the application of the Fenton process for the decolorization of Reactive Black 5 stated that the process is spontaneous but exothermic (Δ*H* = −67.2 kJ mol^−1^) under natural conditions.

Interestingly, the Fenton process is very effective in removing dyes from aqueous solutions. However, in the case of simulated and, even more so, real dyeing wastewater, this efficiency is not always as high. A clear example can be the results of studies on the application of Fenton’s reagent to wastewater from the dyehouse by Ledakowicz et al. [187], who found that the higher the content of NaCl present in textile wastewater, the poorer the decolorization degree, because the rate of oxidation reaction with chloride ion-radicals, which may be formed at high concentrations of Cl^−^, is slower than the reaction of hydroxyl radicals. Moreover, the emulsification effect of surfactants present in textile wastewater at the concentration above the critical micelle concentration causes a decrease in decolorization rate. Simulated textile wastewater is not the same with respect to decolorization by Fenton’s reagent, as real wastewater generated during reactive dyeing in industrial scale. It was proved that nearly a five times bigger reagent dose had to be used to decolorize a mixture simulating the composition of real textile wastewater than in the case of a dyestuff solution without any auxiliary substances, and a nearly seven times higher dose has to be applied to decolorize real wastewater generated in an industrial reactive dyeing process than in the case of a dyestuff aqueous solution.

Heterogeneous Fenton catalysts are emerging as excellent materials for applications related to wastewater purification. The heterogeneous Fenton-like reaction has proved to be attractive, with the production of new catalysts such as iron oxides compounded with graphene oxide, Cu sulfate, and vanadium titanate [188]. Of the many solid catalysts, zero-valent iron turned out to be an effective reducing agent that gives out two electrons in the presence of H_2_O_2_ or O_2_ and forms the Fe^2+^ species responsible for Fenton reaction. Under aerobic conditions, it acts by oxidizing contaminants, while in anaerobic conditions, it acts by reducing them [189].

Fe^0^ + O_2_ + 2H^+^ → Fe^2+^ + H_2_O_2_

Fe^0^ + H_2_O_2_ + 2H^+^ → Fe^2+^ + 2 H_2_O

Fe^2+^ + H_2_O_2_ → Fe^3+^ + **·**OH + OH^−^

Over the past decade, the use of zero-valent iron was demonstrated for treating different varieties of organic and inorganic contaminants, such as dyes [190]. Thomas et al., in their recent review on heterogeneous Fenton catalysts [191], reported that zero-valent iron-based technologies have seen huge interest in both mechanistic studies and field applications for the treatment of numerous pollutants due to the environmental compatibility of iron and versatility of the process towards oxidation or reduction reactions. They believed that more applications will be seen in the future, especially if challenges on dispersibility, longevity, and reactivity control are addressed. This cheap catalyst can considerably decrease the operating cost of the reaction and pave the way for commercial-scale applications of Fenton reaction. Additionally, it was proved to be as effective as classical Fenton reagent in the toxicity removal of azo dye Acid Red 27 towards *Vibrio fisheri* [192].

However, these reactions need H_2_O_2_ or O_3_ to generate the powerful •OH radicals for the destruction of organic pollutants in wastewaters or other external stimulants such as light. A more interesting approach to oxidize and remove organic compounds from wastewater in dark ambient conditions is the use of perovskites—crystalline ceramics with a cubic structure described by the general formula ABO_3_. Chen et al. [188] synthesized a catalyst that was composed of perovskite (the content of Ca and Sr in the A-site of the perovskite structure was varied whilst the B-site was Cu rich) and applied it for the degradation of an azo dye, Orange II. The catalyst proved to be effective for breaking –N=N– bonds from solutions containing low dye concentrations (up to 100 ppm). The degradation kinetics under dark conditions were fast, with up to 80% of the dye being degraded within 10 min. TOC accounted for more than 60% of the carbon, whilst the remainder of the carbon was found to be adsorbed on the surface of the spent catalyst. The application of perovskites in dye removal is an interesting alternative, but it still needs further investigation.

Summarizing this chapter on AOPs, we complied the newest achievements in the last 3 years in the field of ozone-based and Fenton-based reactions in Table 3, highlighting the objects, conditions, and effectivity of the studied processes.

## 5. Biological Processes

Biological methods include the application of bacteria, yeasts, fungi, and microalgae (or their metabolites) in situ or ex situ. Ex situ methods are treatments that involve physical elimination of the polluted material for treatment processes, whereas in situ technologies involve the removal of the contaminants at the site itself. Table 4 represents the list of dyes, implemented biocatalysts, and process effectiveness depicted in the literature appearing in the last three years.

There are two main mechanisms by which microorganisms remove dyes: biosorption and biodegradation [45,123,208]. As the results of the biological dye removal are strongly dependent on the enzymes produced by the organisms, at the beginning of this section, the mechanisms of enzymatic dye degradation will be described.

### 5.1. Enzymes and Microbial Community Action in Dye Degradation

Among the enzymes that were detected during the biodegradation experiments, the most important belong to three groups: laccases, peroxidases and azo reductases. Laccase is a multi-copper phenol oxidase. Laccases are non-specific enzymes that catalyze the one-electron oxidation of substituted aromatic compounds to the corresponding radicals with the concominant reduction of molecular oxygen to water [14]. They are the most often implemented in the degradation of azo dyes. Fungal laccases begin the degradation of azo dyes with the asymmetric cleavage the of –N=N– double bond and oxidative cleavage, desulfonation and deamination subsequently, while the bacterial laccases lead to the formation of the phenolic compounds via the free radical mechanism without cleaving the azo bond [209]. The reason for the observed differences is in the redox potential of the enzymes—the bacterial laccase is defined as a low-redox enzyme [210] and is not able to cleave the azo double bond.

Peroxidase is a heme-containing enzyme that may be of different origins (from microorganisms, plants, and animals) [211]. Among the existing peroxidases, those most often implemented in dye degradation were manganese peroxidase, lignin peroxidase, horseradish peroxidase, and soybean peroxidase. The catalytic cycle of peroxidases involves the formation of two intermediates: Compound I and Compound II, according to the following reactions:

Peroxidase + H_2_O_2_ → Compound I + H_2_O

Compound I + SH → Compound II + S

Compound II + SH → Peroxidase + ^•^S + H_2_O

where SH indicates a generic substrate [212]. Although the peroxidases were successfully implemented in the different dye’s degradation [211], the details concerning the mechanism were revealed only in the case of the azo dye removal. Apparently, the symmetrical azo cleavage was observed with subsequent radical-initiated ring opening of the metabolites [212].

Another important group of enzymes used in the dye degradation are azo reductases [209]. They may be divided into two types: membrane-bound and cytoplasmic. The membrane bound azo reductases cleave the azo bond by transferring electrons to azo dye that acts as the electron acceptor. As a result, azo dye is converted into aromatic amines—colorless but still with potential toxicity [209]. The membrane-bound azo reductases utilize the metabolic products of certain cellular substrates as the redox mediators to act as an electron shuttle [209]. Those enzymes are capable of azo bond cleavage only in anaerobic conditions by the redox potential below −350mV [213]. Further mineralization of formed aromatic amines demands aerobic or at least anoxic conditions [209].

The dyes may adhere to bacterial surfaces through covalent, electrostatic, or molecular forces. Peptidoglycan has been reported to be an important factor that affects the bacterial adsorption of dyes [208]. As a rule, bacterial adsorption does not involve chemical reactions—rather, it is physical adsorption [214]. The adsorbed dyes may be degraded inside the bacterial cell (after the transport through the cell membrane, [155]) or outside the bacterial cells [209]—for example, high molecular weight sulfonated azo dyes are unable to pass through the cell [214]. Bacteria have a diverse and dynamic metabolism. The mechanism of dye degradation by bacteria involves various oxidoreductive enzymes which utilize xenobiotic compounds as substrates and convert them into less complex metabolites [215]. The following enzymes were found in the bacterial cultures treating dyes: flavin reductase, nicotinamide adenine dinucleotide hydrogen-dependent 2,6-dichlorophenol-indophenol (NADH-DCIP) reductase, malachite green (MG) reductase, lignin peroxidase, laccase, tyrosinase, riboflavin reductase, aminopyrine, N-demethylase, veratryl alcohol oxidase, aryl alcohol oxidase and azo reductase [215]. Depending on the culture conditions—anaerobic, anoxic, or aerobic—different enzymes play the most important role in the decomposition of dyes. The utilization of microbial consortia offers significant advantages over the use of pure cultures. Different strains may attack dye molecules at various positions. Moreover, decomposition products, which appeared due to the metabolic activity of one strain, may be used as a substrate by another strain [14].

The fungal dye removal occurs by adsorption on the fungal mycelium in the first step [123]. Depending on the dye structure, different physico-chemical interactions are mainly responsible for the adsorption process [216]. Functional groups such as carboxyl and amino were found to be very important as they were responsible for the electrostatic attraction [216]. Additionally, phosphate groups and the lipid fraction play an important role in dye biosorption. In the case of living cells, the dye degradation takes place as the result of the extracellular nonspecific enzyme activity such as manganese peroxidase, lignin peroxidase, or laccase [217].

In the case of algal dye treatment, the polysaccharides, lipids and proteins present on the surface of algal cell wall have various functional groups, such as hydroxyl, carboxyl, amino, and phosphate, that are considered to be responsible for the sequestration of dyes from wastewater through electrostatic attraction, adsorption, chelation, ion exchange and complexation process [45]. Additionally, Omar [218] stated that a strong attractive force exists between mono-azo dye tetrazine and *Chlorella* cells. As a result, fast diffusion onto the external surface was followed by fast diffusion into the algal cells. Mahajan and Kaushal [139] observed dye in the cytoplasm of macroalgae *Chara vulgaris*. Moreover, from FTIR spectral results they concluded that there were possible electrostatic and hydrogen bond interactions of methyl red dye with active carbonyl and hydroxyl functional groups of the cell wall of macroalgal surface.

Microalgae can be effectively employed to bioremediate textile wastewater (dyes and nutrients removal). Microalgae have risen in prominence, mostly due to their potential for simultaneous bioremediation, CO_2_ mitigation, and also high added-value molecule production (biodiesel) [219,220]. Oyebamiji et al. [140] also looked into textile wastewater treatment in terms of biomass generation, heavy metal reduction and decolorization using six different microalgae strains from Chlorellaceae family. The authors concluded that green microalgae cultured in textile wastewater is a promising and sustainable method for biofuel diesel exploitation. Behl et al. [141] used microalgae coupled with graphene oxide for decolorization of textile wastewater and subsequent lipid production. Their results indicate that the systems removed 90% of the Direct Red 31 (DR 31) dye within 150 min under visible light.

The most common biological method used for the dye containing wastewater treatment is an activated sludge process (ASP). Activated sludge (AS) is a complex biocensosis that consists mainly of bacteria [14]. Additionally, there are also some protozoans present in the AS microflora. The most important feature of ASP is a formation of flocs that may be removed from treated wastewater by a sedimentation process. The backbones to which floc-forming bacteria adhere are filamentous microorganisms [221]. Extracellular polymeric substances (EPS) produced by bacteria or absorbed from wastewater form AS flocs [222] binding together living or dead bacteria, precipitated salts, inorganic particles (e.g., sand) and organic fibers [223]. Apart from rather weak forces at the outer part of the flocs, the interior is stabilized by chemical binding forces in which divalent cations (e.g., Ca^2+^) play an important role [223]. Due to the fact that the bacteria are incorporated into the flocs, the mass transfer phenomena also plays an important role in the kinetics of biodegradation [224]. Disperse, vat, direct, and basic dyes may be easily adsorbed onto flocs that have weak negative charge [225]. Anthraquinone and azo dyes are decomposed by means of the mechanisms described in the paragraph devoted to bacterial dye degradation. Due to the significant biodiversity of AS microflora, the advantages of bacterial consortia are visible.

Biofilms are sticky, viscous, slimy, negatively charged layers of specially structured microbial conglomerations of a single or multiple species that are attached to biotic or abiotic surfaces through embedding in a self-synthesized matrix of extracellular polymeric substances (EPSs) [226]. Bacterial biofilms have many advantages over the free planktonic bacterial species, such as protection from the adverse effects of a changing environment, the ability to exchange nutrient and genetic materials, survival in different metabolic states, and increased tolerance against various toxic compounds, e.g., chemicals, organic pollutants, heavy metals, and antibiotics [227]. The mechanism of dye adsorption by biofilms is complex and results from intraparticle diffusion as well as surface adsorption. The adsorption process may occur in two stages: macropore diffusion (i.e., the transport of dye molecules in the solution to the biofilm surface) and micropore diffusion (i.e., the adsorption of dye molecules to the active sites of biofilm) [228].

Recently, one of the most often investigated bioreactor types is the moving bed biofilm reactor (MBBR). A moving bed biofilm reactor links the advantages of both activated sludge and biofilm [14]. Biomass grows on the support that is in a constant motion. This assures complex microbiota, a lower space requirement, and easier separation between solid and liquid phases typical for biofilms, and the absence of clogging problems [171]. MBBR may work in anaerobic or aerobic conditions [155].

### 5.2. Anaerobic Treatment

Although azo reductases are produced by different organisms—starting from bacteria, ending in higher eucaryotes [229]—the bacterial azo reductase has the highest potential for dye degradation and, as a result, anaerobic treatment is based on bacteria. The anaerobic processes basically demand gas-tight bioreactors in order to prevent oxygen penetration and assure strongly reductive conditions. Moreover, during the anaerobic treatment, biogas might be generated (depending on the organic loads and the presence of compounds toxic for the methanogens), and it must be safely discharged from the bioreactor and utilized. In the laboratory, experiments on the anaerobic processes were often conducted in Erlenmeyer flasks (at stationary mode, [117,118,144]). Furthermore, different bioreactor constructions were used—Upflow Anaerobic Sludge Blanket reactors (UASB, [145,147,230]), anaerobic baffled reactor coupled with downflow hanging sponge (ABR + DHS, [148]), continuous stirred tank reactors (CSTR, [145]), anaerobic dynamic membrane bioreactors (AnDMBR, [143]).

In comparison to aerobic processes, anaerobic processes could save a lot of energy and reduce the need for a post-treatment of excess sludge [144]. However, they may lead to the generation of by-products that are more dangerous for the environment than parent dyes. For example, Punzi et al. [230] observed that after anaerobic treatment of Remazol Red (RR) solutions and textile wastewater in a UASB reactor, acute toxicity towards *Vibrio fisheri* and *Artemia salina* increased. Simultaneously, they confirmed the mutagenicity increase.

The anaerobic conditions are preferable for azo dyes decolorization as they enable azo reductase induction and action (suitable redox potential) leading to complete color removal [215]. The efficiency of azo dyes degradation may be enhanced by the addition of redox mediators [214] or adsorbents serving as carriers for microorganisms [147]. Although the anaerobic degradation of anthraquinone dyes is also possible, there are few literature data concerning the experiments performed in such conditions [143,144,208]. Cai et al. [144] stated that resuscitation-promoting factors may accelerate the induction of enzymes necessary for xenobiotic substance (anthraquinone dyes) degradation and, as a result, improve the efficiency of the process.

The biodegradation kinetics are most often described by the simplest unstructured model developed by Monod. This is based on the assumption that there is one substrate *S* that limits the growth of the microorganisms—according to the Michaelis–Menten model for the enzymatic reaction:

$$\frac{dX}{dt}=\mu_{max}\frac{S}{K_{m}+S}\cdot X$$

where *μ_max_* is the maximum specific growth rate and *K_m_* is the saturation constant for the substrate (Monod constant).

In the case of the substrate concentration changes, some authors used a simple enzymatic reaction model (Michaelis–Menten) without the microbial growth considering:

$$-\frac{dS}{dt}=R_{max}\frac{S}{K_{m}+S}$$

where *R_max_* is the maximum rate of substrate removal (mg L^−1^ min^−1^) [155].

For low concentrations of substrate, the above-mentioned Michaelis–Menten model may be simplified to a first-order reaction model:

$$\frac{dS}{dt}=-k_{1}S=-\frac{R_{max}S}{K_{m}}$$

While for high substrate concentrations to zero-order reaction rate:

$$\frac{dS}{dt}=-k_{0}=-R_{max}$$

Moreover, Castro et al. [155] also used second-order irreversible kinetics:

$$\frac{dS}{dt}=-k_{2}S^{2}$$

Among the other simple models, pseudo-first order kinetics were implemented [213]. Franca et al. [204] considered dyes as xenobiotic substances and decided to apply a model with a sigmoid function that was described as follows:

$$\frac{dS}{dt}\times \frac{1}{X}=\frac{S}{X}\times \frac{ab}{1+e^{-b\left(t-c\right)}}\left(1-\frac{1}{1+e^{-b\left(t-c\right)}}\right)$$

where *S* is the dye concentration (mg L^−1^), *X* is the biomass concentration (mg VSS L^−1^), while *a* (unitless), *b* (h^−1^) and *c* (h) are constants.

Castro et al. performed the kinetic analysis of Reactive Orange 16 (RO16) removal by indigenous biomass in static Erlenmeyer flasks in the presence of co-substrate [155] They adjusted zero, first, and second order reaction models to experimental kinetics and concluded that data followed the second-order kinetics. The increase in co-substrate concentration (from 200 to 400 mgO_2_ L^−1^, measured as COD) led to the decrease in the kinetic rate constant from 0.0177 to 0.0095 L mg^−1^ h^−1^. Another approach to the dye degradation kinetics was made by Franca et al. [117]. They implemented sigmoidal model to Acid Red 14 (AR14) transformation by *Oerskovia paurometabola*. The values of the three model parameters were estimated as a = 33.4 ± 1.3 (unitless), b = 0.21 ± 0.04 h^−1^, and c = 32.3 ± 0.2 h. The authors stated that this model is a useful means of predicting AR14 degradation kinetics by *O. paurometabola* and enables the scaling-up of the process.

The investigations of Thanavel et al. [118] may serve as an example of metabolic pathways of azo dye degradation under anaerobic conditions. They proposed the degradation pathways of three reactive dyes, Remazol Red (RR), Reactive Red 180 (RR 180) and Reactive Black 5 (RB5), caused by a bacterial strain *Aeromonas hydrophila* SK16 on the basis of increased level of enzymes and GC-MS profiling. RR 180, by the action of azo-reductase on azo bonds, was divided into two transitional compounds [A] and [B]. Compound [A] was further desulfonated into an unidentified product. Compound [B] was asymmetrically cleaved into compounds [C] and [D]. Compound [D] was identified as benzamide [Rt 21.093 min; Mol.wt-121; *m*/*z*-123]. Furthermore, desulphonation and deamination of Intermediate [C] led to the formation of naphthalene-2-thiol. The asymmetrical cleavage of azo dyes is a well known action of laccase [211]. In RB 5, initially azo reductase broke azo linkage, leading to the production of Intermediate [I] and [II]. As a result of Intermediate [I] deionization, the 2-[(4-aminophenyl)sulfonyl]ethyl sulfate was formed. Laccase cleaved Intermediate [II], forming Intermediate [III] and [IV]. Intermediate [III] was degraded to 4-dihydronaphthalene-2-sulfonate. Furthermore, desulfonation and deionization of Intermediate [IV] led to the formation of 3,5,6-triamino-3,4-dihydronaphthalene-2-sulfonate. Sequential deamination formed 5-amino-3,4-dihydronaphthalene-2-sulfonate and 3,4-dihydronaphthalene-2-sulfonate as a final product.

Reactive blue 19 (RB19), one of the most widely used anthraquinone dyes, was chosen to show degradation pathways of this dye group. RB19 was degraded in the laboratory scale UASB reactor [144]. GC-MS analysis revealed two main transformation products, i.e., C_8_H_11_NO_6_S_2_ and C_2_H_6_O_7_S_2_. Based on these intermediates, Cai et al. proposed the possible degradation pathway for RB19. The appearance of C_8_H_11_NO_6_S_2_ was assumed to be a proof of the C-N groups cleavage—the aromatic amine structure, which was probably due to hydrolysis in the anaerobic conditions. Moreover, the identification of C_2_H_6_O_7_S_2_, which was probably generated by further degradation of C_8_H_11_NO_6_S_2_, provided evidence for the breakdown of the C-S group.

### 5.3. Aerobic Treatment

Among the microorganisms that are able to produce enzymes necessary for dye degradation under aerobic conditions, white-rot fungi are the most efficient [215,231]. However, experiments were conducted on bacteria (especially *Streptomyces*) [34,119,134], yeast [127], and other fungi [120,124,128,202,203] for the implementation of aerobic dye removal. Although bacterial laccase is less efficient in the azo bond cleavage than fungal laccase, this drawback may be partially overcome by the addition of a natural redox mediator—methyl syringate in 0.5 mM concentration led to the Acid Orange 63 decolorization increase from 0 to 35% [34]. Moreover, bacterial or yeast laccase obtained from halo- or thermophilic microorganisms may act in a wider range of pH and temperatures than white-rot fungi laccase [34,127,134]. However, there is also one literature report concerning the engineering of fungal laccases with a successful shift of optimum catalytic activity to alkaline pH [135]. Other methods of effectiveness improvement are the implementation of a bacterial–fungal consortium [129] or the addition of a co-substrate [202].

Aerobic treatment was investigated with the usage of whole microbial cells [120] as well as isolated enzymes [34]. In the case of whole microbial cells, the experiments were conducted on plates [128], in Erlenmeyer flasks [119,127,130,202], in Erlenmeyer flasks with fungus immobilized in LECA [203], in stirred tank reactor with *Aspergillus niger* immobilized on pieces of *Luffa cylindrica* [124], in a filled column bioreactor [120], and in a packed bed reactor [119], with decolorization efficiency varying between 60 and 97%. As can be seen above, a lot of experiments were conducted with fungi immobilization—this is the result of the morphologic specificity of fungal growth. As for the isolated enzymes implementation, investigations were primarily performed in disposable cuvettes [34,120,134], vials with immobilized peroxidase [132], and Erlenmeyer flasks with free and immobilized laccase [136]. The color removal obtained using free or immobilized enzymes varied between 33 and 98%. Despite these indisputable achievements, there are no applications of enzymes in the treatment of industrial dye wastewater.

The biological treatment under aerobic conditions led to the decrease in the acute microbial toxicity towards: *Pseudokirchneriella subcapitata* [34], *Sinorhizobium meliloti* [127] and *Vibrio fisheri* [136]. Additionally, the phytotoxicity (*Vignia radiata* [120], *Sorghum vulgare* and *Phaseolus mungo* [127], lettuce seeds [136]) and cytotoxicity [203] were reduced.

Mohamed et al. investigated the kinetics of the simultaneous biodegradation of methylene blue and phenol by *Trametes hirsute* [202]. Among the three tested kinetic models (zero, first and second order reactions), the best fitting to the experimental data was obtained for the first-order reactions. The addition of co-substrate increased the values of the kinetic rate constant from 5.8—7.6·10^−3^ to 2.4—3.4·10^−^^2^ L min^−1^. Zhao et al. implemented the Michaelis–Menten kinetic model to the Coomassie brilliant blue G-250 (CBB) removal by extracellular enzymes (laccase, lignin peroxidase LiP and manganese peroxidase MnP) [122]. Based on the kinetic parameters, they stated that lignin peroxidase had the biggest influence on the CBB degradation—the maximum substrate uptake rate for LiP was equal to 30.3 U L^−1^ with a saturation constant of 0.6 mmol L^−1^, while the same parameters for laccase were 0.1 U L^−1^ and 5.4 mmol L^−1^, respectively.

The aerobic degradation pathways of azo dyes were described on the basis of Reactive Black 5 (RB5) transformation by *Sterigmatomyces halophilus* SSA1575 [127]. The asymmetrical reduction of azo bonds of RB5 by the action of NADH-DCIP reductase was found as the first step. This resulted in the formation of amines (2-((4-aminobenezene)sulfonyl)ethoxy)sulfonic acid [a] and 1,2,7-triamino-8-hydroxy -3,6-naphthalinedisulfonate (TAHNDS). TAHNDS, as an unstable compound, might have been rapidly transformed to other smaller intermediate metabolites, such as 2,7,8-triaminonaphthalen-1-ol [b]. Subsequently, [a] and [b] were most probably further oxidized into smaller compounds and may eventually be mineralized. The intermediate metabolite 2,7,8-triaminonaphthalen-1-ol might have been deaminated into naphthalene-1,2,4-triol, and then transformed to catechol, which might be cleaved oxidatively into aliphatic metabolites via the cis-muconic acid pathway followed by the tricarboxylic acids cycle and final mineralization of RB5. Simultaneously, (2-((4-aminobenezene)sulfonyl)ethoxy)sulfonic acid was most probably further oxidized into 2-((4-aminophenyl)sulfonyl)ethanol, 4-ethanesulfonyl aniline, and aniline through desulfonation. Then, the later metabolite was probably deaminated into benzene [127].

For the anthraquinone dyes, the metabolic pathways will be shown on the basis of Anthraquinone violet R (AVR) and Alizarin cyanine green (ACG) treatment by *Myrothecium verrucaria* [120]. ACG was converted into a low molecular compound with molecular formula C_19_H_14_O_3_ and *m*/*z* value 313.0839 and further to C_12_H_11_N and *m*/*z*—170.097. In the case of AVR, two-step reactions occurred—the first compound formed was C_15_H_10_O_3_ with *m*/*z* value 239.0712, followed by C_7_H_3_N_3_O_2_ with m/z value 162.0301. Generally, the first step of bacterial anthraquinone dye degradation involves the dissociation of the small molecular groups around the anthraquinone rings from the parent compound under aerobic conditions. The anthraquinone ring is gradually broken, forming much smaller molecular compounds through oxidation and hydrolysis. Further cleavage of the anthraquinone metabolites led to the formation of small molecules such as benzoic acid [208].

### 5.4. Combined Anaerobic and Aerobic Treatment

**Figure 2:**
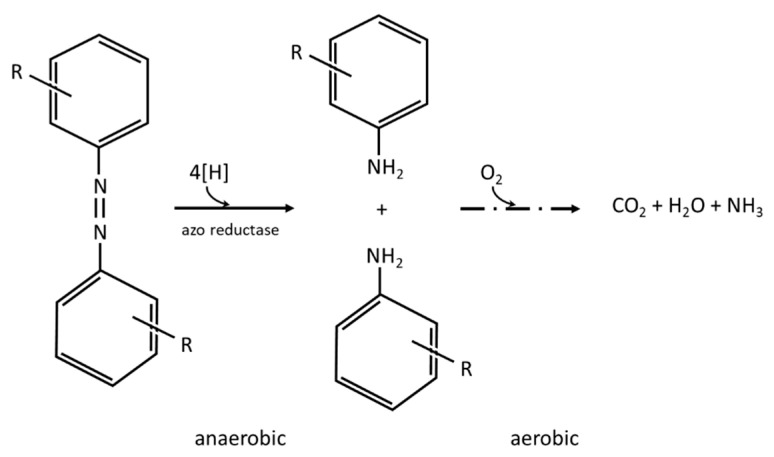
Scheme of azo dyes degradation in two step anaerobic-aerobic treatment.

Azo dye removal and detoxification require the application of alternating anaerobic and aerobic conditions. The aromatic amines produced as a result of azo bonds cleavage in the anaerobic step are further degraded in the aerobic one (Figure 2) [232]. The combined anaerobic–aerobic treatment most often uses mixed bacterial cultures such as activated sludge [149,204,205,207], anaerobic granular sludge [150,153], anaerobic–aerobic granular sludge [142,206], immobilized activated sludge [151], or biofilm [111,153,155]. The sequencing of anaerobic–anoxic–aerobic conditions is easy to obtain in the sequence batch reactors (SBRs, [204,205])—the decolorization efficiency reached 100% by up to 94% of COD removal. However, two-sludge batch systems (anaerobic SBR followed by the aerobic one) assure both decolorization and aromatic amines degradation [233] by the production of a sustainable energy source—biogas [149]. Yan et al. [206] assured the formation of anaerobic–aerobic granules that they further used in aerated batch columns—simultaneously obtaining 88% color and 70% aromatic amine removal. There were also many continuous two-steps systems tested of different constructions, e.g., UASB + stirred tank reactor (STR) with activated sludge [204], UASB with microaeration in the upper part [150], hybrid anaerobic bioreactor + aerobic biofilm reactor [153], or anaerobic + aerobic STRs with immobilized biomass [151]. The increase in the acute toxicity towards *Vibrio fisheri* was observed after the anaerobic stage [150,207] as a result of aromatic amine (AAS) formation. In the case of a very high loads of AAS, the activated sludge was not able to detoxify them [207]. On the contrary, even microaeration was able to assure the toxicity decrease [150]. The phytotoxicity (towards *Triticum aestivum* [151] and *Raphanus sativus* [111]) was completely removed after two-step treatment.

Most often, the description of biodegradation kinetics for two-step systems was divided into anaerobic and aerobic separately using the simple models shown in Section 5.2 [155]. However, Hameed and Ismail [151] also took into account the oxygen limitation in the aerobic biodegradation:

$$\frac{dX}{dt}=\mu_{max}\frac{S}{K_{m}+S}\times \frac{O_{2}}{K_{O_{2}}+O_{2}}\times X$$

where *O*_2_ is the oxygen concentration (mg L^−1^) and *K_O2_* is the half-saturation constant of oxygen (mg L^−1^). Additionally, they considered the effect of the internal mass transfer resistance on the biodegradation activity of immobilized cells, using the effectiveness factor (η). As they obtained an effectiveness factor value equal to 0.9 and 0.99 for the anaerobic and aerobic conditions, respectively, they stated that diffusion had a very slight effect on the biodegradation.

Bahia et al. [207], apart from the previously mentioned models, also used a substrate inhibition model:

$$-\frac{dS}{dt}=\mu_{max}\frac{S}{K_{m}+S+\frac{S^{2}}{K_{i}}}\times X$$

where *K_i_* is the inhibition constant (mg L^−1^). The authors stated that the Michaelis–Menten model fitted the best to the color removal, while the first-order model fitted best to the COD removal.

The aromatic compounds’ appearance during the anaerobic phase and their degradation during the aerobic phase were confirmed (among the others) by Zhu et al. [142]. By means of GC-MS, they detected the presence of benzidine and naphthol at the end of the anaerobic treatment, while small molecular components such as alcohols and acids were detected in the effluent after aerobic conditions.

## 6. Integrated AOPs and Biological Methods

The combination of chemical oxidation and biological processes is most often performed in one of three ways:

1. chemical post-treatment after biodegradation;
2. chemical pretreatment before biodegradation;
3. biodegradation before and after chemical oxidation, possible more than one cycle or integrated system [14].

Among all chemical oxidation processes, ozonation and Fenton reagents are used in the industrial practice [14]. Table 5 represents the list of dyes and implemented biological and advanced oxidation processes with their effectiveness depicted in the literature in the last three years. Only six publications have been found in the literature in the last 3 years.

### 6.1. Chemical Post-Treatment

The most sensible solution seems to be a biological degradation as a first step of a combined system [230]. In this case the biodegradable fraction of wastewater may be degraded by a cheaper and more environmentally friendly method. The chemicals or energy input necessary for the further degradation of the remaining contaminants would be lower than those used for the raw loads [14] and used only for the removal of compounds resistant to biological oxidation [238]. The biological processes are effective in organic load removal—up to an 80% COD decrease [41,230,239]. Additionally, anaerobic biodegradation leads to almost complete decolorization [230]. However, anaerobic processes generate products that may be more toxic than the parent compounds—after anaerobic treatment in a UASB reactor, the Remazol Red (RR, concentrations 100 and 500 mg L^−1^) toxicity towards bioluminescent bacteria *Vibrio fisheri* and the textile effluents toxicity towards shrimp *Artemia salina* increased. Although the subsequent ozonation enabled significant reduction of RR and textile effluent toxicity towards both organisms, the results changed disproportionately according to the ozone dose [230]. Goswami et al. [234] coupled a packed bed bioreactor (PBBR) filled with Arjuna (*Terminalia Arjuna*) seeds biochar immobilized with *Providencia stuartii* with ozonation for the degradation of Congo Red (CR) dye. Although the biological step was aerobic, it led to 92% color removal. Further ozonation enabled complete CR removal. The economic feasibility of the total energy consumption was evaluated as around 3.5 kW of energy for the degradation of 1 g of CR dye. In the case of sequential anaerobic–aerobic biological treatment, Ledakowicz et al. [238] performed an investigation on the combination of SBRs and ozonation in the industrial textile wastewater treatment. They stated that the biological processes led to the substantially higher removal of organic carbon compounds and toxicity than chemical process, while the ozonation process was more effective in decolorization. In turn, catalytic ozonation (with iron shavings) enabled 100% removal of proteins and 42% removal of polysaccharides through a decrease in the inhibitory effect from 51 to 33% in the case of bio-treated dyeing and finishing wastewater polishing [240]. Azizi et al. [241] implemented SBRs (with the external feeding at the beginning of the aeration phase) and an enhanced Fenton (by ultrasound radiation) process in the removal of azo dye Acid Red 18 (AR18). The biological processes enabled over 90% COD and color removal. They also got rid of 36% metabolites generated during the AR18 degradation. Fenton was mainly responsible for the metabolites’ degradation—up to 90%. In the case of full-scale industrial textile wastewater treatment, the Fenton process significantly improved color and COD removal (66 and 73%, respectively) obtained in pure-oxygen activated sludge treatment [242]. Ribeiro et al. [243] tested whether Fenton process may be used as a polishing step enabling water reuse. They stated that additional Fenton treatment assured COD (68% removal), turbidity (88% removal), and other important parameters required for water reuse. Moreover, visual color was comparable to that of fresh water.

The results obtained by Brindha et al. [235] are used as an example of degradation pathways that may occur during the two step biological-chemical dye treatment. Using enzymatic analysis, high resolution liquid chromatograph mass spectrometry (HR-LCMS) analysis, potentiometric titration, photoluminescence (PL) spectra, and electrochemical impedance spectroscopy (EIS), the authors observed Mordant Yellow 10 (MY10) degradation pathways in anaerobic biodegradation by *Pseudomonas aeruginosa* BRPO3 and visible light-driven photo-Fenton oxidation with zero-valent iron Fe^0^. The most probably NADH/FAD-dependent azoreductase, NADH-DCIP reductase, and laccase were responsible for biological decomposition of MY10. Three reactions were proposed: (1) azo (–N=N–) bond cleavage with the formation of 4-amino benzene sulfonic acid and 5-amino salicylic acid, (2) asymmetric cleavage of C10-N9 bond of dye molecule arising benzosulfonic acid and 5-diazine salicylic acid, and (3) reactive desulfonation with production of 5-(phenylazo) salicylic acid that may be further cleaved symmetrically to form 5-amino salicylic acid and aniline. The low TOC removal (19%) confirmed that anaerobic biodegradation products need further degradation. Aromatic amines formed in anaerobic biodegradation were further mineralized in a photo-Fenton process. Initially, two primary reactions took place on the surface of Fe^0^: (1) solid phase and (2) aqueous phase reactions. In a solid phase reaction, H_2_O_2_ is partially adsorbed on the Fe^0^ surface and oxidizes Fe^0^ to Fe^2+^_surf_. Then, partially adsorbed Fe^2+^_surf_ activates H_2_O_2_ to generate **^·^**OH, and is oxidized to Fe^3+^. In the aqueous phase reaction, Fe^0^ loses two electrons to the oxidant H_2_O_2_ in acidic medium to form Fe^2+^ ions and OH^−^ anions. These free Fe^2+^ ions generate **^·^**OH. Fe^3+^ ions generated from both reactions either react with water molecules with the formation of a complex which dissociates into Fe^2+^ and **^·^**OH in the presence of light (photo-Fenton) or enters into a Fenton-like reaction. In a Fenton-like reaction, Fe^3+^ ions react with H_2_O_2_, generating Fe^2+^ and hydroperoxyl radicals (**^·^**O_2_H). The oxidation potential of hydroxyl radicals is stronger than **^·^**O_2_H [244]. In the presence of light, photo-Fenton reactions most probably dominated the entire iron redox cycle, not the Fenton-like reaction. Photo-Fenton degradation of the primary intermediates from the bacterial decolorization of MY10 took place in four phases: C-N cleavage, C-S cleavage, ring opening, and mineralization. The hydroxyl radicals target primarily the electron-rich easily breakable π bonds of the azo groups, followed by the amino group, sulfonates group, and deactivated aromatic rings. As the result of the addition of OH group to the nitrogen atoms present in –N=N– bond in 5-diazine salicylic acid, a compound C_7_H_8_N_2_O_5_ was formed. The hydrogen atom in the amino group of 4-amino benzene sulfonic acid was replaced by an OH group forming 4-hydroxyamino benzenesulfonic acid. Further release of H_2_O most probably formed a nitroso intermediate (not detected during this study) that was attacked by radicals with the formation of 4-nitro benzenesulfonic acid. Then, the hydroxyl group was added to the unsaturated bond of nitroso group radical and was shifted to the oxygen atom. This theoretical intermediate broke up on aryl radicals and nitric acid, in which the former recombined with the OH group to form 4-hydroxy benzenesulfonic acid. In the case of 4-hydroxy benzenesulfonic acid desulfonation (C-S bond cleavage), the initial step is the conjugate addition of OH group at C-5, resulting in carbon radical at C-4 position relative to the sulfonic group, which was quenched by another hydroxyl radical to form geminal hydroxyl sulfonate. This compound further broke up and lost the sulfonic group to produce hydroxyl ketone, which is more prone to an oxidative ring cleavage reaction. The presence of short chain carboxylic acids confirmed the ring cleavage of the formed aromatics. A ring cleavage reaction follows similar patterns: radical attack, elimination, and bond cleavage. As an example, in ketones, **^·^**OH attacks the ketonic group that cleaves the C4-C5 bond to form a carbon radical at the C5 position. The latter is attacked by **^·^**OH to produce dihydroxy compound that fused together to form dicarboxylic acid. Almost 92% of TOC was removed in the photo-Fenton process, confirming that dye intermediates as well as other microbial metabolites were mineralized to a great extent [235].

### 6.2. Chemical Pre-Treatment

If dyes or dye-loaded wastewater are completely non-biodegradable, then chemical processes should be used at the beginning of the system. Gottschalk and coauthors recommended chemical oxidation before biodegradation for wastewater for which the BOD_5_/COD ratio is below 0.2 [23]. Although chemical pretreatment is applied as an method of biodegradability improvement, it is also possible that by-products of dye degradation via chemical oxidation may inhibit some metabolic pathways of microorganisms used in the biological post-treatment [35]. This is one of the reasons for the implementation of the toxicity measurements as one of the parameters that should be used for process control. Nevertheless, ozonation is able to increase the BOD_5_/COD ratio—even from 0 to 0.8 [27,245]. The goal of chemical pretreatment is a partial oxidation of dyes—instead of complete mineralization. That also leads to the lowering of chemical and energy consumption (in comparison to the usage of chemical oxidation as a one-step dye treatment) [245].

Fahmi et al. [246] stated that ozonation transforms the functional groups in azo dye into more biodegradable by-products that were further easily removed by the biodegradation. Apparently, they used a Upflow Anaerobic Sludge Blanket (UASB) reactor as the biological step of dye transformation. The authors showed more detailed discussion on the COD removal in their second paper [247]. The first ozonation removed 7.4% of COD and improved the solution biodegradability to such an extent that in the UASB reactor, 29.6% removal of COD was observed. They repeated the cycle and as a result of the second ozonation and second biodegradation, 65.5% of COD was removed.

Dias and coworkers [35] made an attempt to identify the by-products of azo dye Reactive Red 239 (RR 239) ozonation and their influence on the subsequent biological degradation in moving bed biofilm reactors (MBBR, two in series). As they observed increasing ozone consumption after total color removal from the solution containing RR 239 and low COD and DOC removal, they stated that it indicates the generation of stable by-products. The ozonation is capable of breaking the azo bond, degrading of naphthalene ring and aromatic sulfonate, but it has difficulties in the opening of the triazine ring. Further degradation was possible via biological processes—they were able to remove up to 90% of the COD. The authors stressed that ozonation products were amenable for heterotrophs but inhibiting for nitrifiers. In similar investigations, Castro et al. studied degradation of Reactive Orange 16 (RO 16) by ozone and, subsequently, by biomass in MBBR [155]. They also observed fast discoloration (97%) due to chemical oxidation by comparatively low COD (50—75%) and TOC (35—40%) removals. The mentioned experiments were also focused on the degradation pathways of RO16.

Recently, the Fenton process has rarely been used as a pre-treatment method in two-step textile dye degradation. Liu et al. [236] performed investigations on the electrode modifications for the electro-Fenton process followed by aerobic granular sludge treatment. They obtained almost complete discoloration of methylene blue after 60 min of EF with above 70% TOC removal. The biodegradation enabled further TOC abatement—up to 86.5%. Shanmugam et al. [237] investigated a combination of a Fenton process with biological treatment (by a defined bacterial consortium) of effluents containing toxic azo dye Acid Blue 113 (AB113) [237]. The initial mineralization of AB113 synthetic solution by the Fenton process resulted in the presence of aromatic amines with smaller molecular weights. The most abundant were benzene acetic acid, diethyl phthalate and n-hexadecanoic acid. Further biological treatment led to the formation of compounds that do not pose a threat for the environment. The most common were benzoic acid, 4-ethoxy-, ethyl ester, pyrrolo [1,2-a]pyrazine-1,4-dione, hexahydro-3 (2methylpropyl) and hexadecanoic acid. The Fenton-treated dye bath effluent showed the presence of a broader range of compounds such as naphthalene, phthalic anhydride, phenol, 3, 5-bis (1, 1-dimethylethyl), phthalic acid, butyl hept-4-yl ester, phenol, 4, 4′-methylenebis. The final treated dye bath effluent showed the presence of more biodegradable alkenes and hydrocarbons (dodecane, tetradecane, hexadecane, heptadecane). It must be stressed that the Fenton process resulted in only 30—40% removal of dye, and bacteria inhibition was observed by the higher dye concentration (1–1.4 g·L^−1^). The dyehouse effluent had to be diluted six times in order to diminish the biomass inhibition [237].

### 6.3. Influence of AOPs Pre-Treatment on Biodegradation Kinetics

There are very few papers dealing with the influence of chemical oxidation on the kinetics of the bioprocesses [248,249,250]. Scott J.P. and Ollis D.F. [251] already pointed out in 1997 that the general parameters, such as BOD or COD, widely employed to measure poorly characterized industrial wastewater, were included in the kinetic models describing parts of AOPs/biological treatment. These models did not properly describe wastewater degradation due to the lack of specificity of the parameters measured, although they are very useful. In the case of wastewater treatment by the activated sludge process, the basic measure of substrates is COD, while the biomass content is estimated on the basis of volatile solids concentration. Considering the previous assumption, the specific substrate removal rate can be expressed by:

$$q=q_{max}\frac{\left[COD\right]}{K_{m}+\left[COD\right]}$$

where *q_max_* is the maximum substrate removal rate (mgO_2_ (mg VSS h)^−1^).

Ledakowicz et al. [249] investigated the influence of ozonation, UV, O_3_/UV and H_2_O_2_/UV processes combined with acclimated activated sludge on the biodegradation kinetics. They stated that the preoxidation led to a faster biodegradation—the maximum specific rate of substrate elimination increased from 0.04 for untreated textile wastewater to 0.07 mg O_2_ (mg VSS h)^−^^1^ after H_2_O_2_ treatment—and that pre-treated pollutants were more available for biological oxidation (significant decrease in the Monod’s constant from 3378 to 759 mg O_2_ L^−1^ for the ozonation and to 323 mg O_2_ L^−1^ for the combined treatment with H_2_O_2_/UV).

Karahan et al. [250] evaluated the effect of ozonation on COD fractionation and kinetic coefficients defining major biological processes. Using oxygen uptake rate tests, they determined the maximum specific growth rate and saturation constant. They observed the influence of ozonation on the remaining organic carbon composition but without the significant changes in the biodegradation kinetics coefficients.

### 6.4. Pilot and Industrial Plants

Following on from our considerations, the only feasible option for the industrial implementation of textile wastewater treatment is to use chemical oxidation methods as a pre-treatment and biological as a post-treatment, or vice versa. Most of the studies cited so far concerned laboratory, bench-scale studies. The literature review on scaling-up provides only a few publications, concerning pilot plants and even less on industrial implementations of integrated biological processes and AOPs.

Bilińska et al. [252] tried to scale-up the ozonation process, focusing on the investigation of the accumulation of by-products in a multi-recycling system. The brine that was produced from dyeing wastewater treated by ozonation in a 20 L bubble column reactor after 30 min was completely decolorized and recycled successfully. The authors concluded that ozonation can be applied in the industry as a method for textile wastewater recycling.

A pilot-scale reactor of almost 3 m^3^ effective volume was built and run by Ma et al. [253] to investigate the removal of organic pollutants in bio-treated dyeing wastewater by heterogeneous catalytic ozonation with waste iron shavings as a catalyst. The process in the pilot scale was effective in the removal of pollutants from wastewater so that the limit of direct discharge (<80 mg L^−1^ of COD) was met. The catalyst was stable and effective in its properties and was recommended as a good choice for heterogeneous catalytic ozonation.

Sen et al. [254] investigated biological degradation of the mono azo dye, methyl orange (MO) contained in textile effluents from a site in the Raman Textiles, Khurda, Odisha, India, in a fabricated polyethylene batch reactor with 500 L of working volume with coconut fiber as a support for biofilm development. The bioreactor inoculated with *Pseudomonas putida* (MTCC-1149) from twenty-four-hour old cultures of pure isolates was operated in suspended growth with micro-aerophilic microenvironment with a cycle period of 48 h. Supernatant exchange of 90% was employed where 10% of biomass was retained in the bioreactor for use in subsequent cycle operations; 87% decolorization was observed after 90 h at all tested concentrations, while the COD removal was 69%. This decolorization efficiency required peptone and glucose, which play a pivotal role in the decolorization activated by the enzymatic reduction of azo bonds. The study showed that the induction of micro-aerophilic micro-environment might have helped the simultaneous reduction/oxidation reactions. When analyzing the textile wastewater subjected to decolorization, it should be noted that it was a neutral (pH 7.6), very low loaded (21.8 mg L^−1^ COD), easily biodegradable (BOD_5_/COD = 0.58) effluent, hence the good bio-discoloration and biodegradation were obtained.

An interesting combination of the activated sludge process and filtration (membrane bioreactor MBR) integrated with ozonation and photocatalysis was developed in pilot scale to treat the real textile wastewater by Sathya et al. [255]. This ozonized MBR had the working volume of 20 L, with a ozone dosage rate of 1–5 g h^−1^ and Hydraulic retention Time (HRT) 2–5 h, while the photochemical reactor with a 5 L working volume with a visible lamp at the top, centered, was filled with tungsten oxide photocatalyst immobilized onto spongy alginate beads (500 mg L^−1^). The biodegradability efficiency was enhanced from BOD_5_/COD = 0.27 to 0.58, COD removal was 93%, and TOC decreased from 309 to 62 mg L^−1^. Photocatalysis, as a post-MBR step, provided a complete removal of color from textile wastewater but was explored only on single dye removal, while the color removal of real textile wastewater was 94%. The treated wastewater met the discharge norms prescribed by the Indian statutory body in terms of COD, color, and suspended solids.

Bae et al. [242], characterizing the refractory matters in dyeing wastewater discharged from 61 dyeing factories in Ansan, South Korea, reported on a full-scale treatment plant (100,000 m^3^ d^−1^) where the wastewater after screening and settling was neutralized, equalized, and treated in the pure-oxygen activated sludge with HRT of 6h, and then in the Fenton oxidation unit (HRT 0.5h; pH 3.5; H_2_O_2_ 4mM, FeSO4∙7H_2_O 4.2 mM). The Fenton oxidation effluent was coagulated for 15 min at pH 6.0 followed by clarification and discharged to a public sewer flowing into a municipal wastewater treatment plant. It appeared that the Fenton process merely impaired the color-imparting bonds instead of completely degrading them. This process significantly reduced the soluble COD (66%) and color (73%) remaining after initial biological treatment, which reduced SCOD by 53% and color by 13% in raw wastewater.

A full-scale plant with a treatment capacity of 400,000 m^3^ d^−1^ by Chen et al. [182] was designed and run continuously to treat the effluents of bio-treated dyeing and finishing wastewater using Fenton oxidation in 16 pipeline reactors, each of 6.9 m^3^ volume. After 24 s of reaction time in the 6.9 m^3^ pipeline reactor, the Fenton reaction was terminated by increasing the pH to above 6. The COD decreased from 140 to 77 mg L^−1^, (removal efficiency of 45%) and DOC from 35 to 26 mg L^1^. The kinetics of Fenton decolorization was studied using a stopped-flow spectrophotometer at second-scale intervals and soluble COD and DOC in the real biologically treated wastewater in a batch test during Fenton oxidation. Although the time predicted from the kinetic model was much longer, approx. 2.7 min, which was caused by different mixing conditions in the compared scales, this is nevertheless an excellent example of using kinetic studies to design a reactor on an industrial scale.

Ozonation has been used in the textile industry for many years in the treatment of dyeing wastewater; however, there is little information in the literature on this subject—only occasionally can you find information on upgraded ozone generators, such as in a wastewater plant in the Como region, Italy [256]. De Nora supplied a complete system consisting of two ozone generators (max. capacity of 600 kg O_3_ d^−1^ each) with a liquid oxygen feed system and porous diffusers to optimize O_3_ transfer efficiency. In addition to removing color from wastewater, the new system addresses additional treatment needs including COD for sludge reduction and disinfection before discharge to the Como river.

In Biliński Textile Company, Poland, there is a wastewater treatment system coupling biodegradation in anoxic/oxic activated sludge process and ozonation in order to recycle a low-loaded wastewater stream as process water [14]. The final stage of the treatment is ozonation carried out by injecting ozone with the concentration of 144 gO_3_ m^−3^ during 10 min. Complete decolorization of biologically treated wastewater is carried out by means of an industrial ozonation installation built by Thies Company, Germany. The total volume of bubble columns for the ozone reaction is 25 m^3^. The installation is equipped with an ozone generator with a capacity of 2.5 kg O_3_ h^−1^ [257].

**Figure 3:**
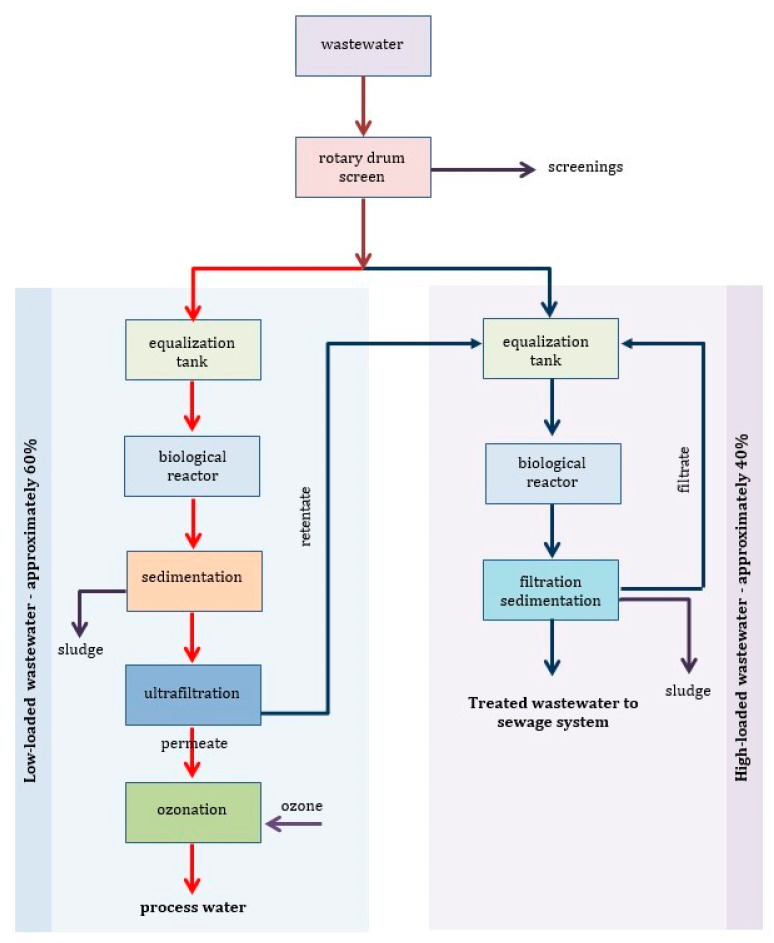
The block diagram of the technology proposed within the ICBTOS project.

Within the project ICBTOS (no. PBS2/A9/22/2013 financed by the National Centre for Research and Development), the investigations were performed on a similar system, combining biological processes and ozonation in the treatment of both wastewater streams separately (low-loaded and high-loaded). The implementation of horizontal continuous flow bioreactor (HCFB, 10 L working volume) or biological aerated filters (BAFs, 15 L working volume) filled with ceramsite enabled 60% COD, 70% toxicity, and 50% color removal from high-loaded wastewater stream. Subsequent ozonation led to further organics (up to 80%), color (up to 90%), and toxicity (up to 92%) decrease. However, from the economic point of view, single biodegradation of the high-loaded stream is a good enough alternative for the flocculation/coagulation process used so far (for instance in Biliński Textile Company, [14]). As a result, the technology shown in Figure 3 was proposed—biodegradation, ultrafiltration, and ozonation for the recycling of the low-loaded wastewater stream, and biodegradation for the utilization of the high-loaded wastewater stream together with the retentate after ultrafiltration of the low-loaded stream. Nevertheless, this system may be used only in the case of high-loaded wastewater with a BOD_5_/COD ratio above 0.2. For non-biodegradable wastewater, the conventional flocculation/coagulation process (low-cost option) or ozonation pre-treatment (high-cost option) should be implemented.

## 7. Conclusions and Future Perspectives

As seen in this review, intensive research has been carried out in the area of separate chemical oxidation and biodegradation of dyes as well as integrated processes combining both methods. The mechanisms of ozone-based chemical oxidation with or without the participation of catalysts or H_2_O_2_ have been presented in detail. In addition, the Fenton reaction mechanism was described. Unfortunately, the kinetic models of these oxidation processes rarely reflect the presented mechanisms of oxidation reactions, but rather roughly treat the rate of decolorization by means of pseudo-first order reaction kinetics.

Similar conclusions could be drawn regarding the biological degradation of dyes. The mechanisms of biodegradation were investigated in detail, including metabolic pathways of intermediates. However, the kinetics of these processes leave much to be further studied, especially in the combined processes of AOPs and biodegradation. Furthermore, competition for chemical oxidants by the contaminants and multiple substrate and co-substrate kinetics for biological mixed cultures should be studied. The mechanisms and kinetics of the degradation of individual dyes were most often investigated, whereas, in industrial effluents, there are multiple substrates, and therefore decolorization and mineralization of wastewater should be examined, considering many dyes, and also with the presence of additives and high concentrations of salt, which may cause inhibition of biodegradation.

Only a few studies have provided treatment costs, and therefore researchers should concentrate on the cost analysis of the newly developed methods, because no one will propose the implementation of a new photochemical method of wastewater decolorization without cost analysis, especially without the recently required life cycle assessment (LCA) method. The same applies to the membrane filtration technique, which, however, was not reviewed here due to lack of space. The cost of membrane filtration limits its application—this is true in lab-scale studies, hence this method is rarely used in the large scale.

Despite the fact that the ozonation of industrial wastewater has been used for many years on an industrial scale, except for in a few cases, there are no reports of the use of catalysts in this process on an industrial scale. The Fenton reaction, especially with the use of new heterogeneous catalysts, has a good chance of industrial application, but it requires testing on a pilot scale and further increasing the technological readiness level (TRL) of this process.

Future studies should focus on the cost-effectiveness of ozone-based AOPs integrated with biological degradation, as the economically viable techniques, to carry out more pilot plant experiments with real industrial wastewater, and further process optimization and scaling-up.

## Figures and Tables

**Table 1:** Typical parameters of wastewater streams generated during reactive dyeing of cotton [27].

Effluent	pH (−)	Conductivity (mS cm^−1^)	Cl^−^ (g L^−1^)	COD (mgO_2_ L^−1^)	BOD_5_ (mgO_2_ L^−1^)	BOD_5_/ COD (−)	TN ^1^ (mg L^−1^)	TP ^2^ (mg L^−1^)
washing	9.9–10.0	2.25–4.9	0.30–0.74	2080–2440	535–620	0.25–0.26	2.3–78	1.8–8.0
acidification	4.4–7.1	1.19–2.4	0.08–0.11	750–2300	200–250	0.11–0.27	1.0–38	0.4–9.4
rinsing	4.5–7.0	0.77–1.46	0.06–0.08	240–1280	80–95	0.07–0.33	0.5–17	0.1–6.9
dyeing	10.2–11.6	61.6–110	23.3–35.1	350–3710	25–75	0.02–0.07	22–25	0.5–8.5
rinsing	10.6–10.8	13.7–25.5	7.38–9.55	235–1075	30–40	0.04–0.13	8.8–10	0.1–3.0
acidification	3.6–8.7	2.69–11.3	2.58–3.29	360–1160	60–280	0.17–0.24	3.8–6.8	0.1 –4.0
washing after dyeing	5.5–8.8	0.30–5.45	0.80–1.46	505–965	40–400	0.08–0.40	3.6–11	0.4–0.9
rinsing	6.8–9.1	0.05–1.63	0.27–0.58	280–550	70–300	0.25–0.55	2.0–5.3	0.1–0.4
rinsing	7.6–8.7	0.03–1.35	0.12–0.39	155–185	50–130	0.32–0.71	1.4–2.2	0.02–0.2
neutralisation	5.4–7.9	0.06–1.07	0.06–0.13	120–470	35–200	0.29–0.43	0.5–1.2	0.21–0.49
final mixed effluent	9.6–9.9	12.4–12.6	3.6–4.9	960–970	170–240	0.25	5.5–18.7	0.5–4.5

^1^ total nitrogen, ^2^ total phosphorus.

**Table 2:** Current development in processes used in dye removal.

Process	Current Development	References
Adsorption	Synthesizing of new, efficient, nature-based, or waste-originating adsorbents, kinetic, equilibrium and thermodynamic studies on biosorption	[42,43,44,45,46]
Coagulation/flocculation	Synthesizing of new, efficient, nature-based, or waste-originating coagulants and acceleration of sedimentation by magnetic field	[47,48,49,50,51,52]
Electro-coagulation	Ultra-sound assistance, nanofilms on cathodes and solar power usage	[53,54,55,56,57,58]
Electrochemical oxidation	Air-diffusion cathodes, new materials and coatings of electrodes, membrane anode and electro-peroxone process	[59,60,61,62,63,64]
Membrane filtration	Novel membrane materials, with addition of graphene, stabilization of membranes by biomacromolecules	[65,66,67,68,69,70]
Ozonation	Catalyst addition, enhancement by ultrasound and hydrodynamic cavitation	[71,72,73,74,75]
O_3_/UV	Photocatalytic membranes	[76]
O_3_/H_2_O_2_	Proposal of the degradation mechanism, enhancement by electrolysis, heterogenous catalyst addition	[77,78,79]
UV/H_2_O_2_	Measurements of cytotoxicity, mutagenicity and phytotoxicity changes, proposal of degradation mechanism, comparison of different UV sources	[80,81,82,83,84,85]
Photocatalytic oxidation	Synthesizing nanoparticles, efficient under visible light or difunctional catalysts, green methods of catalyst synthesis	[86,87,88,89,90,91]
Fenton	Fenton-like heterogenous catalysts enabling dye degradation in a wide range of pH, among the others zero-valent iron catalysts, green or one-spot synthesis of catalysts, fixed bed reactor application, implementation of sulphate radical anions	[92,93,94,95,96]
Photo-Fenton	Fenton-like heterogenous catalysts enabling dye degradation under visible light, waste-originating catalysts, proposal of degradation mechanism	[97,98,99,100,101,102]
Electro-Fenton	Kinetics and cost analysis, synthesizing of nanocomposite electrodes, air-diffusion cathode, proposal of mechanism and degradation pathways, novel orbiting electrodes reactor and recirculation flow-through reactor	[103,104,105,106,107,108]
Bacterial treatment	Isolation of new strains or consortia from activated sludge, oxidation ditch, palm oil mill effluent or desert soil, alkali-, halo- and thermophilic strains implementation, consortium with algae, bacteria immobilization, co-substrate addition, proposal of mechanisms, pathways genome and transcriptome analysis	[109,110,111,112,113,114,115,116,117,118,119]
Fungal treatment	Implementation of microbial consortium (e.g., yeast consortium with ability of lignin valorization dye treatment and biodiesel production), fungi immobilization, isolation of new strains from plant roots or effluent site	[120,121,122,123,124,125,126,127,128,129,130]
Enzyme treatment	Optimization of enzyme production, enzyme immobilization, metabolites and toxicity assessment	[131,132,133,134,135,136]
Algal treatment	Immobilization, co-contaminant influence on dye biodegradation, genetic modification of algae and cyanobacteria, graphene oxide addition and lipid production	[137,138,139,140,141]
Activated sludge, anaerobic sludge	Granule formation (anaerobic core with aerobic shell), metagenomic analysis in anaerobic MBR, addition of resuscitation-promoting factors, integration of anaerobic and aerobic reactors, addition of halotolerant yeast and magnetic field	[142,143,144,145,146,147,148,149,150]
Biofilm	Application of new biocarriers, co-substrate addition, kinetic analysis and process optimization in moving bed biofilm reactor, biomass acclimatization and optimization of anoxic/aerobic sequencing batch moving bed bioreactors	[151,152,153,154,155]

**Table 3:** Brief summary of dye removal using selected advanced oxidation processes (last three years).

Object	Catalyst	Conditions	Effectiveness	Reference
**Ozonation**				
Reactive Orange 16, Reactive Red 120, Direct Red 80	none	Found as optimal: pH 11, time 10 min, initial dye concentration 2000 mg L^−1^	52–64% dye removal	[74]
Direct Red 81	none	Found as optimal: pH 11, time 27 min, initial dye concentration 2000 mg L^−1^	61% dye removal	[193]
Acid Black 1	none	Found as optimal: ozone concentration 70 mg L^−1^, pH 6, time 20 min, initial dye concentration 200 mg L^−1^	95.5% decolorization	[167]
Direct Red 80	none	Ozone dosage 1.25 g L^−1^ min^−1^, pH 2–13.5 (optimal 12), initial dye concentration 100 mg L^−1^	99% decolorization, 27.4% COD removal	[77]
Reactive Red 239	none	Ozone concentration 20 mg L^−1^, pH 7, time 20 min, ozone consumption 106.9 mgO_3_ L^−1^, initial dye concentration 50 mg L^−1^	100% decolorization, 62% COD and 35% DOC removals	[35]
Acid Red 14	none	pH 10.7 (tested also 6.65), time 25 min, initial dye concentration 1500 mg L^−1^	93% decolorization	[194]
Crystal Violet	none	Ozone dose 2 mg L^−1^ min^−1^, pH 6.8, time 60 min, initial dye concentration 50 mg L^−1^	78% decolorization	[79]
Direct Black 22	none	Ozone dose 5 g h^−1^, pH 3–11 (optimal 11), time 30 min	55% COD removal	[195]
Rinsing effluents containing Reactive Blue 19, Reactive Red 239, Reactive Yellow 176	none	Ozone concentration 20 mg L^−1^, gas flow rate 0.5 L min^−1^, pH 7	60% COD removal after 60 min	[196]
Effluents after electrocoagulation containing mainly Reactive Black 5	none	Transferred ozone dose 600 mgO_3_ L^−1^, pH 11, time 30 min,	Above 90% decolorization	[75]
**Catalytic Ozonation**				
Methyl Orange	Ni-based layered double hydroxides (Ni-LDHs) nanomaterials 1–3 g L^−1^	Ozone flow rate 109 mg h^−1^, pH 9, initial dye concentration 100–500 mg L^−1^	96% decolorization, 72% COD removal	[197]
Direct Black 22	Zinc slag 0.125–1 g L^−1^	Ozone dose 5 g h^−1^, pH 3–11 (optimal 11), time 30 min, 100 mg L^−1^ H_2_O_2_ addition	69% COD removal, 76% COD removal with H_2_O_2_	[195]
Reactive Blue 194	Activated carbon	Ozone concentration 178.8 mg L^−1^, time 40 min, pH 5–12, 25–50 °C, NaCl addition 5–50 g L^−1^	100% decolorization, up to 90% COD removal	[198]
Reactive Black 5	Silver-cobalt composite oxide 0.2–1 g L^−1^	Gas flow rate 30 L h^−1^, pH 2.2–12, initial dye concentration 100–1000 ppm	Up to 99% TOC removal	[72]
Alizarin Red S	activated carbon powder/c-Fe2O3 nano-composite 0.2–2 g L^−1^	Ozone dose 5 g h^−1^, pH 3–11, time 30 min, initial dye concentration 100–500 mg L^−1^	Up to 95% dye removal, 40% mineralization	[199]
Rinsing effluents containing Reactive Blue 19, Reactive Red 239, Reactive Yellow 176	MnFe_2_O_4_@CA 1 g L^−1^	Ozone concentration 20 mg L^−1^, gas flow rate 0.5 L min^−1^, pH 7	100% decolorization after 15 min, 65% COD removal after 40 min	[196]
Effluents after electrocoagulation containing mainly Reactive Black 5	Activated carbon 0.5 g L^−1^	Transferred ozone dose 500 mgO_3_ L^−1^, pH 11, time 30 min,	Above 90% decolorization, toxicity decrease	[75]
Textile wastewater	Copper-doped zinc oxide 1–4 g L^−1^	Ozone flow 10 to 40 g m^−3^, time 30 min, pH 3–11 (optimal 7)	Up to 90% COD removal	[176]
**Peroxone**				
Crystal Violet	none	Ozone dose 2 mg L^−1^ min^−1^, pH 3–9 (optimal 9), time 30 min, initial dye concentration 25–200 mg L^−1^	100% decolorization	[79]
Direct Red 80	none	Ozone dose 1.25 g L^−1^ min^−1^, H_2_O_2_:O_3_ ratio equal to 1:10500, pH 2–13.5 (optimal 13), initial dye concentration 100 mg L^−1^	99% decolorization, 43% COD removal	[77]
Direct Black 22	none	Ozone dose 5 g h^−1^, pH 3–11 (optimal 11), time 30 min, 100 mg L^−1^ H_2_O_2_ addition	66% COD removal	[195]
**Fenton Based**				
Methylene Blue	iron (II) sulphate 2–5 mM	10–80 mM H_2_O_2_, 20–40 °C, pH 2–7 (optimal 3), initial dye concentration 10–50 ppm, time 30 min	98.8% decolorization, 85% COD removal	[183]
Acid Yellow 17	iron (II) sulphate 0.04–0.07 mM	0.50–1.1 mM H_2_O_2_, 25 °C, pH 2–8 (optimal 3), initial dye concentration 0.06–0.09 mM, time 60 min	89% degradation	[184]
Acid Red 27	iron (II) sulphate 60–100 mg L^−1^	20–100 mgH_2_O_2_ L^−1^, pH 2–5 (optimal 3.5), time 30 min, initial dye concentration 100 mg L^−1^	72% COD removal	[192]
Ponceau Xylidine, calconcarboxylic acid	iron (II) sulphate 1.7 mM	7.3 mM H_2_O_2_ or Na_2_S_2_O_8_,	Decolorization: 94% (1 min), 100% (240 min), COD removal (240 min): 40% Fe/HP, 60% Fe/SPS	[95]
Methylene Blue	pulverized *Malacantha alnifolia* tree bark modified with iron (II) sulfate 0.5–1 g L^−1^	Fe^2+^/H_2_O_2_ ratios: 10/10,000–100/10,000, pH 2–8, time 60 min	97% dye removal	[96]
Acid Red 27	Zero valent iron 60–100 mg L^−1^	20–100 mgH_2_O_2_ L^−1^, pH 2–5 (optimal 3), time 30 min, initial dye concentration 100 mg L^−1^	69% COD removal, toxicity decrease	[192]
Crystal Violet	zerovalent iron nanoparticles dispersed on polyester fabrics 5 g L^−1^	20–100 mLH_2_O_2_ L^−1^ (optimal 100), pH 5–9 (optimal 5)	99% dye removal	[92]
Methyl Orange	iron (III) nanoparticles synthesised with *Dimocarpus longan* extract 0–18.5 mg L^−1^	0–46.8 mM H_2_O_2_, time 30 min, pH 2–10 (optimal 3), initial dye concentration 50–250 mg L^−1^	100% dye removal	[200]
Methylene Blue	iron (III) loaded on activated carbon (2–15 wt%)	Packed bed reactor, 0.0163–0.326 M H_2_O_2_, pH 2–9 (optimal 3.5), temperature 30–50 °C, initial dye concentration 100 mg L^−1^	70% dye removal	[94]
Eriochrome Black, Methylene Blue, Rhodamine B	core-shell nickel cobalt spinel coated with iron phthalocyanine 4–6 g L^−1^	40–60 mM H_2_O_2_, pH 4–6.7, initial dye concentration 5–20 mg L^−1^	100% dye removal, 90% TOC removal (Rhodamine B)	[93]
Orange II	calcium strontium copper loaded perovskite 1 g L^−1^	Without H_2_O_2_, time 90 min, initial dye concentration 10–100 ppm	95% degradation, 40% carbon removal	[188]
Methyl Orange	Perovskite 10–25 mg L^−1^	Without H_2_O_2_, pH 2–10 (optimal 2.5), time 20 min, initial dye concentration 20 ppm	90% degradation	[201]

**Table 4:** Brief summary of dyes removal using biological processes (last three years).

Object	Catalyst	Conditions	Effectiveness	Reference
**Bacteria**				
Reactive Red 120	*Pseudomonas guariconensis*	Inoculum: 2% (*w*/*v*) (2 g) of immobilized (alginate beads) effective isolate in MSM broth + 0.5% glucose, temperature 28 ± 2 °C, initial dye concentration 100 mg/L^−1^	91% dye uptake	[114]
Brilliant Crocein	*Providencia rettgeri*	Oligotrophic medium, 2% *v/v* inoculum, time 8 d, 500 mg L^−1^ ethanol, initial dye concentration 50 mg L^−1^	100% dye removal	[115]
Direct Black G	*Anoxybacillus sp.* PDR2	10% *v/v* inoculum, microaerobic conditions, time 48 h, initial dye concentration 100–600 mg L^−1^	82–98% decolorization	[116]
Reactive Black 5, Brilliant Violet 5R, Reactive Orange 16	*Halomonas* sp	Shaken Erlenmeyer flasks, pH 6–11, 2 10% salinity, initial dye concentration 50 mg L^−1^	Above 90% decolorization: RB5–24 h, RV5–13 h, RO16–3 h	[119]
Reactive Black 5 and cadmium	*Pseudomonas aeruginosa* strain Gb30	LB medium, pH 8, 5% *v/v* inoculum, temperature 37 °C, initial concentrations: 0.629 mM Cd^2+^ and 50 mgRB5 ^L−1^	100% decolorization	[112]
Reactive Orange 16, Reactive Blue 250	*Bacillus* sp. VITAKB20 and *Lysinibacillus* sp. KPB6	Different broths: LB, nutrient ZZ, MSM, temperature 37 °C, immobilization in alginate beads, shaking conditions	97.5% RO16 and 98.2% RB 250 degradations	[113]
Reactive Yellow 174 added to industrial textile wastewater	Bacterial consortium: *Sphingomonas paucimobilis*, *Pseudomonas putida* and *Lactobacillus acidophilus*	10% inoculum, temperature 35 °C, time 5 d, initial dye concentration 750 ppm	86% COD removal, 90% decolorization	[109]
Methanil Yellow G	Bacterial consortium: *Halomonas* (49.8%), *Marinobacter* (30.7%) and *Clostridiisalibacter* (19.2%)	1 g L^−1^ yeast extract, pH 10, 10% salinity, temperature 40 °C, time 16 h, initial dye concentration 100 mg L^−1^	93 % decolorization	[110]
Acid Red 14	*Oerskovia paurometabola*	Synthetic textile wastewater containing Emsize E1 (sizing agent) and 1 g L^−1^ yeast extract, anaerobic conditions, temperature 30 °C, initial dye concentration 20–100 mg/L^−1^	91% decolorization	[117]
Reactive Black 5, Brilliant Violet 5R, Reactive Orange 16	*Halomonas* sp	Aerobic PBR, volcanic rock filling, temperature 38 °C, HRT 9–11 h, initial dye concentration 50–150 mg/L^−1^	95% RO16, 79.5% RV5 and 81% RB5 removals	[119]
Congo Red	*Bacillus* sp. MH587030.1	MBBR, polyurethane foam-polypropylene carrier, pH 5–9, carrier filling ratio 10–60% *v/v* initial dye concentration 10–100 mg/L^−1^	Dye removal up to 95.7%	[38]
**Fungi**				
Cotton Blue, Crystal Violet, Malachite Green and Methyl Violet	*Bjerkandera adusta*	4 g inoculum, shaking 150 rpm, initial dye concentration 50–250 mg/L^−1^	Above 90% decolorization	[123]
Reactive Red, Reactive Yellow F3R, Black Cell, Navy VB, Red RB, Remazol Yellow RR, Turquoise	*Neurospora* sp	10% *v/v* inoculum, 2% glucose, 0.1% yeast extract, static conditions, pH 8, temperature 30 °C, time 5 d, initial dye concentration 100–200 mg/L^−1^	48–89% decolorization	[125]
Methylene Blue and phenol	*Trametes hirsuta*	MGY medium, shaking 150 rpm, pH 7, time 6 h, phenol initial concentrations: dye 25–100 mg/L^−1^, phenol 10–50 mg/L^−1^	80% removal of MB and phenol within 80 min	[202]
Remazol Brilliant Blue R, Methyl Orange and Methyl Red	*Aspergillus iizukae* EAN605	5% *v/v* inoculum, shaking 150 rpm, room temperature, time 9 d, initial dye concentration 100–1200 mg/L^−1^	Up to 95% RBBR, 85% MO and 48%MR removal	[126]
Reactive Black 5, Azure B, Reactive Red 120, Reactive Blue 19, Acid Scarlet GR	*Sterigmatomyces* *halophilus* SSA1575	10% *v/v* inoculum, different carbon and nitrogen sources, static conditions, temperature 30 °C, time 24 h, initial dye concentration 50 mg/L^−1^	51–83% decolorization of dye mixtures	[127]
Congo Red	*Aspergillus flavus* JKSC-7	Minimal medium, shaking 120 rpm, temperature 25 °C, time 3 d, initial dye concentration 25–200 mg/L^−1^	97% decolorization	[128]
Coomassie Brilliant Blue	*Lactarius deliciosus*	LCM, shaking 120 rpm, pH 5, temperature 28 °C, time 12 h, initial dye concentration 25–800 mg/L^−1^	99% decolorization	[122]
Reactive Black 5, Reactive Red 120, Reactive Blue 19, Reactive Green 19, Bromophenol Blue, Azure B, Methylene Blue, Methyl Red, Malachite Green, Congo Red, and Scarlet GR	Yeast consortium: *Meyerozyma guilliermondii*, *Yarrowia* sp. and *Sterigmatomyces halophilus*	Basal medium with different carbon sources, time 24 h, temperature 18 °C, initial dye concentration 100 mg/L^−1^	56–81% decolorization of mixtures, 100% RR120 decolorization, fatty acids production observed–biofuels	[121]
Synozol Red HF–6BN and Synozol Black B	Soil borne fungi: SN12f and SN13a isolates	Minimal media, shaking 120 rpm, temperature 28 °C, time 5 d, initial dye concentration 50–250 mg/L^−1^	80–95% decolorization	[130]
Reactive Orange 16	*Pleurotus ostreatus* and *Candida* *zeylanoides*	*Pleurotus ostreatus* colonizing polyamide carrier, shaking 80 rpm, temperature 28 °C, time 11 d, initial dye concentration 25–200 mg/L^−1^	87.5% decolorization	[129]
Congo Red	*Aspergillus terreus* QMS-1	Immobilized on *Luffa cylindrica*, in aerobic stirred tank reactor, 1% glucose, 1% ammonium sulphate, time 24 h, pH 5, initial dye concentration 100 mg/L^−1^	92% dye removal	[124]
Anthraquinone Violet R, Alizarin Cyanine Green	*Myrothecium verrucaria* ITCC-8447	Column reactor with fungi immobilized on Scotch-Brite^®^ or *Luffa cylindrica* support, time 24 h, temperature 30 °C, initial dye concentration 50 mg/L^−1^	80% ACG and 60% AVR decolorization	[120]
Reactive Blue 4, Reactive Blue 19, Acid Blue 29	*Trametes hirsuta* D7	Immobilized on activated LECA, shaking 100 rpm, temperature 30 °C, 1% *w/v* co-substrate, initial dye concentration 100 mg/L^−1^	90% RB 4, 95% RB 19 and 96% AB 29 degradation	[203]
**Algae**				
Methyl Red	*Chara* *vulgaris* L.	20–50 g L^−1^ algae, pH 3.5–9.5, time 48 h, initial dye concentration 10–50 mg/L^−1^	70–100% decolorization	[139]
Textile wastewater	Chlorellaceae family	13% *v/v* inoculum, shaking 120 rpm, constant illumination, 0.5–2% wastewater	Heavy metals and chromogenic substances concentration decrease	[204]
Disperse Blue 1, Disperse Orange 3	bacterial-algal consortium	Moving bed anaerobic bioreactor (38 °C) + photobioreactor (25 °C), LED lamps 402 ± 8 μmol m^−2 s−1^, 12:12 h light/dark, HRT 8 d	78% TOC, 47% nitrogen and 26% phosphorus removals, 96–99% decolorization	[111]
Methyl Red and Congo Red	*Scenedesmus obliquus* free or immobilized in alginate	20% *v/v* inoculum or 100 alga beads/100 mL, temperature 25 °C, time 10 d, initial dye concentration 20 ppm	55% MR and 62% CR decolorization by alga beads	[137]
Malachite green	genetically engineered *Synechococcus elongatus* PCC 7942	Fed-batch process, 10 × 10^6^ cells mL^−1^, temperature 30 °C, time 12 h, constant light 100 μmol m^−2^ s^−1^, initial dye concentration 100 mg/L^−1^	99.5% dye removal	[138]
Direct Red 31	graphene oxide–*Desmodesmus sp.* bionanocomposite	1 g L^−1^ bionanocomposite, 500 W halogen lamp (constant illumination), time 150 min, initial dye concentration 40 mg/L^−1^	92% decolorization, lipid production	[205]
**Enzymes**				
Anthraquinone Violet R, Alizarin Cyanine Green	Crude laccase from *Myrothecium verrucaria* ITCC-8447	1.51 U mL^−1^, pH 3–11 (optimal 9), time 10 min, temperature 20–50 °C, initial dye concentration 50 ppm	56–63% ACG and 52–60% AVR removal	[120]
Malachite Green	Crude laccase from *Trametes versicolor*	40–200 U L^−1^, pH 4.5, shaking 150 rpm, temperature 25 °C, time 60 min, initial dye concentration 100 ppm	95% decolorization	[133]
Reactive Blue 19, Basic Violet 4, Methyl Violet, Methyl Green, Methylene Blue, Poly R-478, Congo Red	Crude enzymatic extract from *Phanerochaete chrysosporium* CDBB 686	0.2 g mL^−1^ enzymatic extract, temperature 35–45 °C, time 12–36 h, H_2_O_2_ concentrations 0.5–1.5 mM, initial dye concentration 50 ppm	42% CR, 57% Poly R-478 and 70% MG decolorization	[131]
Methyl Orange, Methyl Red, Bromocresol Green, Bromothymol Blue, Bromophenol Blue, Coomassie Blue R250 and Phenol Red	Horseradish peroxidase immobilized onto a functionalized reduced graphene oxide-SiO	pH 7, temperature 25 °C, time 60 min, initial dye concentration 200 ppm	100% decolorization for most dyes	[132]
Ponceau 2R, Methyl Orange, Malachite Green, Gentian Violet, Reactive Blue 19, Indigo Carmine.	purified laccase from the thermophilic bacterial strain *Thermus* sp. 2.9	0.15 U mL^−1^, addition of redox mediators, pH 5–9, time 6 and 24 h, temperature 60 °C, initial dye absorbance 1.0 units	20–100% decolorization	[134]
Indigo dye	mutant laccase	40–200 mU mL^−1^, 20–300 μM redox mediators, pH 4.5–9, time 2 h, temperature 20–80 °C, initial dye concentration 200 μM	91% decolorization	[135]
Reactive Blue 19	laccase from *Oudemansiella canarii* free and immobilized using the crosslinked enzyme aggregate	0.1 U mL^−1^ (free or immobilized laccase), pH 5, time 24 h, temperature 30 °C, shaking 100 rpm initial dye concentration 100 ppm	100% decolorization	[136]
**Sludge**				
Acid Orange 7, Methyl Orange, Congo Red	anaerobic-aerobic sludge granules	SBR, 48 h cycle time, initial dye concentration 50 ppm	100% decolorization, 90% COD and TOC removals	[142]
Reactive Blue 19	anaerobic sludge	AnDMBR, temperature 37 °C, HRT 5 or 2.5 h, OLR 1–5 gCOD L^−1^, initial dye concentration up to 1 g/L^−1^	97.5% decolorization, 98.5% COD removal	[143]
Basic red 46	activated sludge	SBR, HRT 8 to 24 h, glucose 1 g L^−1^, initial dye concentration 5–500 mg/L^−1^	Up to 100% decolorization, 65–90% COD removal	[204]
Nylosan Yellow E2RL SGR	activated sludge	SBR, HRT 96 h, glucose 6–8 mg L^−1^, initial dye concentration 20–80 mg/L^−1^	85% decolorization and 91% COD removal	[205]
Mordant Orange 1	Anaerobic-aerobic sludge granules	Batch column reactor, 5 g L^−1^ biomass, DO 1 mg L^−1^, glucose 3 g L^−1^, yeast extract 1 g L^−1^, initial dye concentration 20–100 mg/L^−1^	88% dye and 70% aromatic amines removal, 61% mineralization	[206]
Yellow Gold Remazol	anaerobic and aerobic sludges or algae from eutrophicated natural pond	UASB (HRT 24 h) + aerobic activated sludge reactor (HRT 8 h) or UASB (HRT 24 h) + shallow polishing pond (HRT 66 h), residual yeast biomass as nutrients source, initial dye concentration 50 mg/L^−1^	23% dye and 85% COD removal	[207]
Acid Red 88	anaerobic and aerobic sludges	UASB (37 °C) + aerobic reactor (35 °C), synthetic wastewater, HRT 3–24 h (optimal 6 h), initial dye concentration 0.1 g/L^−1^	95% decolorization, 80% COD removal, methane production	[145]
Acid Red B	Co-culture of activated sludge and yeast *Candida tropicalis* A1 and *Pichia occidentalis* A2	Static magnetic field 24.6–305 mT, glucose 2 g L^−1^, yeast extract 1 g L^−1^, time 18 h, initial dye concentration 0.1 g/L^−1^	99% decolorization, 96% COD removal	[146]
Textile wastewater containing Basic Red 46	Anaerobic sludge + Fe_3_O_4_/sludge carbon	UASB, temperature 37 °C, HRT 24 h, increasing TWW volume fraction up to 80%, addition of dye up to 400 mg/L^−1^	97.6% decolorization, 78% COD removal	[147]
Hellozol HSR Reactive Black	anaerobic sludge and biofilm	ABR + DHS, temperature 30 °C, HRT 23.2 d, initial COD 260 mg/L^−1^	58% decolorization, 90% COD removal	[148]
Cibacron Yellow, Cibacron Blue and Methylene Blue	Anaerobic and aerobic sludges	AnSBR (HRT 48 h) + SBR (HRT 6 h), pH 6.8–7.2, initial dye concentration 15 mg/L^−1^	80% decolorization, 99.5% COD removal	[149]
Industrial textile wastewater	Anaerobic and aerobic sludges	AnSBR (HRT 48 h) + SBR (HRT 6 h), pH 6.8–7.2	44% decolorization, 98% COD removal	[149]
Direct Black 22	Anaerobic sludge	UASB with microaeration in the upper part 0.18 mgO_2_ L^−1^, initial dye concentration 0.06 mM	69–79% decolorization, 59–78% COD removal, oxidation of aromatic amines	[150]
**Biofilm**				
Reactive Yellow 15	activated sludge as a source for biofilm	Anaerobic (HRT 30 h) + aerobic SBMBBR *, carriers: sodium alginate, starch or gelatin crosslinked by polivinyl alcohol, initial dye concentration 10–40 mg/L^−1^	100% dye and –100% COD removals	[151]
Reactive Red 2, Reactive Blue 4 and Reactive Yellow 15	activated sludge as a source for biofilm	Carrier: *Orchis mascula* powder cross-linked with polyvinyl alcohol, anaerobic and aerobic conditions in Erlenmeyer flasks, initial dye concentration 10–40 mg/L^−1^	100% decolorization 81–100% COD removal	[152]
Acid Orange 7 added to domestic wastewater	anaerobic sludge and aerobic biofilm	Hybrid anaerobic reactor (with bioelectrochemical system 0.5 V) + aerobic biofilm reactor with recirculation between reactors, granular graphite carrier, DO 2–4 mgO_2_ L^−1^, initial dye concentration 200 or 800 mg L^−1^	97.5% decolorization and 89% COD removal	[153]
Reactive Orange 16	activated sludge as a source for biofilm	SBMBBR *, biocarrier K1 filling ratio 5%, co-substrate concentration 500 mg/L^−1^, initial dye concentration 10–300 mg/L^−1^	89–100% decolorization, 50–97% COD removal	[154]
Reactive Orange 16	activated sludge as a source for biofilm	Anaerobic MBBR, AnoxKaldnes K1 carrier filling ratio 40% *v*/*v*, HRT 6 h, magnetic stirring, co-substrate concentration 400 or 800 mg/L^−1^, initial dye concentration 5 or 25 mg/L^−1^	Up to 61% dye and 92% COD removals	[155]

* SBMBBR—sequence batch moving bed bioreactor.

**Table 5:** Brief summary of dyes removal using combined processes (last three years).

Object	Biological Process	AOPs	Effectiveness	Reference
**Chemical post-treatment**				
Congo Red	PBBR containing *Terminalia Arjuna* seeds biochar immobilized with *Providencia stuartii*	Ozonation	100% decolorization	[234]
Mordant Yellow 10	*Pseudomonas aeroginosa* BRPO3 in static culture	Zero-iron valent photo-Fenton	100% decolorization (biological), 100% aromatic amines removal (chemical), 100% cytotoxicity removal (chemical)	[235]
**Chemical pre-treatment**				
Reactive Red 239	Two MBBRs in series	Ozonation	100% decolorization (ozonation), 90% COD removal (biological)	[35]
Reactive Orange 16	MBBR	Ozonation	97% decolorization (ozonation), 92% COD removal (biological)	[155]
Methylene Blue	Aerobic granular sludge	Heterogenous electro-Fenton	100% decolorization, 86.5% TOC and 75.7% COD removals	[236]
Acid Blue 113, industrial textile wastewater	Bacterial consortium: *Pseudomonas aeruginosa*, *Bacillus flexus* and *Staphylococcus lentus*	Fenton	85% decolorization AB 113, 90% decolorization and 94% COD removal ITW	[237]
